# LncRNA xist regulates sepsis associated neuroinflammation in the periventricular white matter of CLP rats by miR-122-5p/PKCη Axis

**DOI:** 10.3389/fimmu.2023.1225482

**Published:** 2023-12-05

**Authors:** Huifang Wang, Yichen Li, Shuqi Jiang, Nan Liu, Qiuping Zhou, Qian Li, Zhuo Chen, Yiyan Lin, Chunbo Chen, Yiyu Deng

**Affiliations:** ^1^ Department of Intensive Care Medicine, Guangdong Provincial People’s Hospital (Guangdong Academy of Medical Sciences), Southern Medical University, Guangzhou, China; ^2^ Department of Critical Care Medicine, Guangdong Provincial People’s Hospital, School of Medicine South China University of Technology, Guangzhou, China; ^3^ The Second School of Clinical Medicine, Southern Medical University, Guangzhou, China

**Keywords:** lncRNA XIST, miR-122-5p, sepsis, microglia, astrocytes, sepsis associated neuroinflammation, intracranial infection

## Abstract

**Background:**

Neuroinflammation is a common feature of many neurological diseases, and remains crucial for disease progression and prognosis. Activation of microglia and astrocytes can lead to neuroinflammation. However, little is known about the role of lncRNA xist and miR-122-5p in the pathogenesis of sepsis-associated neuroinflammation (SAN). This study aims to investigate the role of lncRNA xist and miR-122-5p in the pathogenesis of SAN.

**Methods:**

Levels of miR-122-5p and proinflammatory mediators were detected in the cerebrospinal fluid (CSF) of patients with intracranial infection (ICI) by ELISA and qRT-PCR. miRNA expression in the periventricular white matter (PWM) in rats was analyzed by high-throughput sequencing. Levels of lncRNA xist, miR-122-5p and proinflammatory mediators in the PWM were measured using qRT-PCR and western blot. Bioinformatics analysis was used to predict the upstream and downstream of miR-122-5p. The interaction between miR-122-5p and its target protein was validated using luciferase reporter assay. BV2 and astrocytes were used to detect the expression of lncRNA xist, miR-122-5p.

**Results:**

The level of miR-122-5p was significantly decreased in the CSF of ICI patients, while the expression of IL-1β and TNF-α were significantly upregulated. Furthermore, it was found that the expression of IL-1β and TNF-α were negatively correlated with the level of miR-122-5p. A high-throughput sequencing analysis showed that miR-122-5p expression was downregulated with 1.5-fold changes in the PWM of CLP rats compared with sham group. Bioinformatics analysis found that lncRNA xist and PKCη were the upstream and downstream target genes of miR-122-5p, respectively. The identified lncRNA xist and PKCη were significantly increased in the PWM of CLP rats. Overexpression of miR-122-5p or knockdown of lncRNA xist could significantly downregulate the level of PKCη and proinflammatory mediators from activated microglia and astrocytes. Meanwhile, *in vitro* investigation showed that silencing lncRNA xist or PKCη or enhancing the expression of miR-122-5p could obviously inhibit the release of proinflammatory mediators in activated BV2 cells and astrocytes.

**Conclusion:**

LncRNA xist could regulate microglia and astrocytes activation in the PWM of CLP rats via miR-122-5p/PKCη axis, further mediating sepsis associated neuroinflammation.

## 1 Introduction

Sepsis-associated encephalopathy (SAE) is the most common type of encephalopathy encountered in patients with sepsis in the intensive care unit (1, 2). Patients with SAE is characterized by symptoms of delirium, coma, and long-term cognitive dysfunction (3). The pathophysiology of SAE is complex and involves several mechanisms, including neuroinflammation, ischemic processes, neurotransmitter imbalances, endothelial dysfunction and mitochondrial dysfunction. Neuroinflammation is a common feature of SAE, which is mediated by cytokines, chemokines, reactive oxygen species. These mediators are mainly produced by activated microglia and astrocytes, endothelial cells, and leukocyte recruitment (4, 5). Microglia and astrocytes are the main inflammatory cells in the central nervous system (CNS), which play a crucial role in the pathogenesis of sepsis-associated neuroinflammation (SAN) (6, 7). The different activation status of microglia serves different functions. There are two phenotypes of microglia: M1 (neurotoxic) and M2 (neuroprotective) phenotype (8, 9). In parallel with microglia, astrocytes can be divided into pro-inflammatory (A1) and anti-inflammatory (A2) phenotype according to its polarization status (10). LPS-activated microglia could induce a shift in the astrocyte phenotype from neuroprotective to neurotoxic profile (A1-polarized astrocytes). A study has reported that activated microglia produced IL-1α, TNF-α and C1q, which would induce the transformation of astrocytes into A1 phenotype (11). The pro-inflammatory phenotypes of microglia and astrocytes were identified as a potential culprit, which lead to SAN (2, 3). Currently, there are few methods to prevent or treat for SAN because its mechanism still remains unclear. Therefore, it is very important for treating SAN to investigate the mechanisms of microglia and astrocytes activation.

Recent studies have shown that long non-coding RNAs (lncRNAs) and microRNAs (miRNAs) play critical roles in the pathogenesis and progression of sepsis associated organ dysfunction (12–14). LncRNA and miRNAs are being studied as biomarkers, which are used to evaluate brain injury severity and predict outcomes from sepsis, stroke, traumatic brain injury, encephalopathy, and delirium (15). Therefore, targeting these non-coding RNAs would hold great potential for the treatment of SAN. NF-κB is closely related to inflammasome activation, pyroptosis, and apoptosis. Down-regulated levels of miR-223 and miR-146a were found in patients with severe sepsis, and miR-223 is directly involved in inflammation by regulating NF-κB signal pathway (14–16). A study has shown that decreased level of miR-122-5p attenuates lipopolysaccharide-induced acute lung injury (16). In addition, miR-122-5p can be identified as a biomarker to predict prognosis of some diseases including cancers, cardiovascular events, sepsis, and nervous system diseases (17–19). Recently, it has been reported that lncRNAs play important functions in CNS (20). X-inactive-specific transcript (XIST) is one of the firstly discovered lncRNAs with prominent roles in the process of X inactivation (21–24). In addition to interacting with chromatin modifying molecules, XIST also can be served as a molecular sponge for miRNAs to modulate expression of miRNA target protein (25). Its roles have been previously elucidated in many diseases, including cancers, ischemic stroke, and inflammatory sickness. However, the role of lncRNA xist and miR-122-5p in the pathogenesis of SAN has not been fully elucidated. This study sought to explore the mechanism of lncRNA xist and miR-122-5p in microglia and astrocytes activation in SAN. Expression of proinflammatory mediators including TNF-α and IL-1β in microglia and astrocytes through activated lncRNA xist/miR-122-5p/PKCη pathway was first examined. We report here that microglia and astrocytes are activated to produce proinflammatory mediators through lncRNA xist/miR-122-5p/PKCη pathway, which may contribute to pathogenesis of SAE by activating neuroinflammation in the PWM of septic rats.

## 2 Materials and methods

### 2.1 Study approval

The clinical study protocol was approved by the ethics committee at Guangdong provincial People’s Hospital (approval NO. KY-Q-2022-176-02), and the participants or their legally authorized representatives provided written informed consent to participate in this study. All animal procedures were conducted in strict accordance with the Animal Protocol: Reporting of *in vivo* Experiments guidelines. Animal ethics were approved by the Guangdong provincial People’s Hospital (approval ID: SYXK2012-0081).

### 2.2 Patient data

This study consecutively recruited 12 patients with intracranial infection (ICI) and 4 patients with non-ICI at Guangdong provincial People’s Hospital Intensive care medicine between January 2021 and December 2021. All patients included in this study must met the following criteria: (a) diagnosed as ICI or non-ICI based on the biochemical and routine tests or bacterial culture of cerebrospinal fluid (CSF); (b) age above 18 years; (c) consent to participate in the study. The exclusion criteria were as follows: (a) died within 24 hours after admission; (b) complicated with tumors; (c) patients with intracerebral hemorrhages or unspecified diseases; (d) pregnant woman.

After enrollment, demographic characteristics and clinical features of the ICI and non-ICI patients were recorded. Demographic characteristics included age, gender, ICU stay time, ventilator duration, APACHE II, SOFA, GCS, Nervous system positive signs, Seizures, MODS. Biochemical indexes included white blood cell (WBC), Procalcitonin (PCT), C-reactive protein (CRP), serum creatinine (Scr), albumin (Alb), bilirubin (BIL), Sputum culture, Urine culture, Cerebrospinal fluid routine and Cerebrospinal fluid biochemistry. The levels of TNF-α and IL-1β in CSF were measured by enzyme-linked immunosorbent assay (ELISA). The level of miR-122-5p in CSF were detected by real-time quantitative polymerase chain reaction (qPCR). The demographic and clinical characteristics of 12 patients with ICI and 4 non-ICI patients enrolled in the study are provided in **Tables 1**, **2**.

**Table 1:** The sociodemographic and clinical characteristics for ICI and non-ICI patients.

Variables	Total n=16	non-ICI n=4	ICI n=12	*p*
Age, Mean ± SD	57(49.7, 69.7)	44(28, 55.75)	55(53.75, 73.75)	0.52
Gender(male%)	5(31.25%)	3(75%)	2(16.6%)	0.067
ICU stay time, median (range)	8(0.75, 13.75)	4.5(0, 10.75)	13.5(10, 26)	0.048*
Ventilator duration, median (range)	12(3.75, 13.75)	12.5(3.75, 26)	10(3.75, 15)	0.808
APACHE II score, Mean ± SD	20.5(12.3,30)	30(15, 34.5)	18(12.25, 26.25)	0.214
SOFA, Mean ± SD	5.5(4.25,7)	5.5(4.25, 8.25)	6(2, 7)	1.000
GCS, Mean ± SD	4(3,7.25)	4.5(2.5, 12.5)	4(3, 7.25)	0.808
Nervous system positive signs, N%	2(16.7%)	1(25%)	1(8.33%)	0.576
Seizures, N%	1(6.25%)	0(0%)	1(8.33%)	0.667
MODS, N%	2(16.7%)	1(25%)	1(8.33%)	0.576

APACHE II, acute physiological and chronic health evaluation II; IQR, interquartile range; ICI, intracranial infections; SD, standard deviation; SOFA, sequential organ failure assessment; MODS, Multiple Organ Dysfunction Syndrome. *p<0.05, there was statistical significance between non-ICI group and ICI group.

**Table 2:** The laboratory parameters of ICI and non-ICI patients.

Variables	Total n=16	non-ICI n=4	ICI n=12	*p*
WBC (×10^9^/L)	9.22(6.17, 17.4)	9.22(4.76, 15.89)	10.58(6.1, 17.9)	0.808
PCT (ng/mL)	0.67(0.45, 1.13)	0.81(0.21, 7.1)	0.67(0.46, 0.96)	0.933
CRP (mg/L)	35(7.7, 123.7)	74.25(7.5, 162.6)	5(7.7, 80.45)	0.57
BIL (mmol/L)	11.5(8.1, 15)	16.8(9.2, 22.22)	9.75(7.42, 14.74)	0.214
Cr (mg/dL)	57.6(47.66, 250.74)	250.89(25, 521)	57.66(47.6, 84.84)	0.683
BUN (ng/mL)	10.32(6.6, 23.87)	22.7(7.07, 43.37)	10(5.7, 15.1)	0.461
Alb(g/L)	35.32(30.72, 38.49)	30.95(26.9.37.4)	36.5(32.88, 40.05)	0.214
Sputum culture, N%	7(43.75%)	4(100%)	3(25%)	0.071
Urine culture, N%	2(12.5%)	0(0%)	2(16.7%)	0.424
Cerebrospinal fluid routine				
Pandy’s test, N%	6(50%)	0(0%)	6(50%)	0.03*
WBC (10^6^/L)	7(1.25, 123.25)	1.5(1, 2.75)	60.5(5.5, 4570)	0.028*
Cerebrospinal fluid biochemistry				
Glu (mmol/L)	3.86(3.23, 5.7)	4.94(3.75, 6.35)	3.64(3.1, 5.11)	0.283
Cl (mmol/L)	136(128.1, 141.37)	131.2(126.7, 139.5)	139(129.3, 141.4)	0.570
Microalbumin	571(409.2, 2538.5)	399(317, 411.5)	1897.5(569, 5991)	0.016*
Cerebrospinal fluid culture, N%	1(6.25%)	0(0%)	1(6.25%)	0.667

WBC, white blood cell; CRP, C-reactive protein; PCT, procalcitonin; BIL, bilirubin; Scr, serum creatinine; BUN, Blood Urea Nitrogen; ALB, albumin. * p <0.05, there was statistical significance between non-ICI group and ICI group.

### 2.3 Animals

Adult male Sprague Dawley (SD) rats (150-200g) were purchased from the model animal research center of Southern Medical University (Guangzhou, China). All animals were housed under a constant temperature and humidity and a 12-h light/12-h dark cycle, with the lights on at 7:00am. Food and water were available ad libitum. All experimental animals were fed according to the ARRIVE guidelines.

### 2.4 Animal model of sepsis

The cecal ligation and puncture (CLP) surgical procedure in rats was performed as previously described to establish the animal model of SAN (26). The rats were randomly divided into two groups: sham and CLP (**Supplementary Figure 1A**). For the CLP group, following anesthesia with an intraperitoneal injection of 2% pentobarbital sodium (30 mg/kg), the abdominal cavity was opened via a midline laparotomy. The cecum was exposed and tightly ligated in the middle portion with a 3-0 silk suture, then perforated 3 times with an 18-gauge needle (small amount of feces was exposed). After being rehabilitated the cecum, the abdomen was closed with a 2-0 silk suture. The rats were resuscitated by injecting pre-warmed normal saline (37°C, 5ml/100g) subcutaneously. To minimize heterogeneity between different experiments, the CLP procedure was always performed by the same investigator. For the sham group, briefly, only open the abdomen to find the cecum, then close the abdomen. The rats in CLP group were sacrificed at 6 and 24 h, 3d and 7d after operation. The chart of animal model of sepsis was displayed in **Supplementary Figure 1A**.

### 2.5 Library preparation and sequencing

To explore whether there are differences in the level of miRNAs expression, the PWM of septic rats at 24h after CLP operation and corresponding control were obtained. The sequencing libraries of miRNAs were performed using BGI platform, and the 150 bp paired-end reads were generated.

### 2.6 Microinjection of miR-122-5p adeno-associated virus into ICV

To investigate whether miR-122-5p is involved in SAN, the miR-NC AAV-GFP or the miR-122-5p AAV-GFP with 4.2×10^9^ viral genomes/µl (original concentration: C_0 = _5.6×10^9^ viral genomes/µl; HEYUAN, contract number. H20706) were microinjected into the left lateral ventricle of SD rats (150-200g) using the following microinjection coordinates: anteroposterior, 0.5 mm; lateral, 1.0 mm; and ventral, 4.0 mm. To evaluate the effect of AAV miR-122-5p on the neuroinflammation in septic brain, at 14 days after the microinjection of the miR-NC AAV-GFP or the miR-122-5p AAV-GFP, the rats were subjected to CLP or sham operation, then divided into 4 groups: Sham, CLP, AAV miR-NC + CLP, and AAV miR-122-5p +CLP. The rats in each group were sacrificed at 24h after CLP or sham operation. Groups of animals with miR-122-5p AAV microinjection were displayed in **Supplementary Figure 1B**.

### 2.7 Microinjection of lncRNA xist Antisense oligonucleotide into ICV

To explore whether lncRNA xist is closely involved in the pathogenesis of SAN, the ASO of lncRNA xist was used to inject into the left lateral ventricle of rats. Either the lncRNA-NC ASO-GFP or the lncRNA-xist ASO-GFP (20 μl; QIAGEN) was microinjected into the left lateral ventricle of SD rats using the following microinjection coordinates: anteroposterior, 0.5 mm; lateral, 1.0 mm; and ventral, 4.0 mm. At 3 days after the ASO microinjection, the rats were subjected to CLP or sham operation, then divided into 4 groups: Sham, CLP, ASO lncRNA-NC + CLP, and lncRNA-xist ASO +CLP. The rats in each group were sacrificed at 24h after CLP or sham operation. Groups of animals with lncRNA xist ASOs microinjection were displayed in **Supplementary Figure 1C**.

### 2.8 Primary astrocytes culture

Primary astrocytes were obtained from postnatal (P1) SD rat. The rat brains were quickly removed and placed in ice-cold PBS. After the membranes and large blood vessels were removed, the dissected brain cortices were placed in medium supplemented with PBS. The brain tissues were digested with trypsin-EDTA. Subsequently, the cells were planted on poly-L-lysine (PLL) precoated cell culture flasks containing DMEM supplemented with fetal bovine serum. The cultures were maintained in a humidified incubator (37°C, 5% CO2). The medium was changed every 3-4 days. After 7-10 days, to remove the microglial cells from the mixed cells, the culture flasks were shaken at 180 rpm at 37°C for 1 h. Fresh culture medium was added, then continued to shake at 250 rpm for 18-20 h to ensure oligodendrocyte isolation. Finally, the astrocytes were harvested by trypsinization. The purified astrocytes (5 ×10^5^ cells per well) were plated on a 6-multiwell culture dish with PLL and cultured in DMEM/F12/FBS medium for 3 days, which prepared for different experiments.

### 2.9 BV2 cells culture

BV2 cells, microglia cell line, purchased from the Pricell Life Science and the certificate number is PC-H2021041401. Cells were routinely maintained in DMEM supplemented with 10% foetal bovine serum (FBS) and 1% penicillin/streptomycin in a humidified atmosphere at 37°C and 5% CO2. BV2 cells were incubated for different experiments at 24h after seeding.

### 2.10 Primary astrocytes and BV2 cells were divided into three groups:

#### 2.10.1 Group I

To study the effect of miR-122-5p on inflammatory cytokines release, purified astrocytes/BV2 cells were digested and seeded in the same number in medium at 5% CO2 and 95% air at 37°C. The intervention was performed when the cell density reached 70%. Firstly, to determine the expression of miR-122-5p in astrocytes/BV2 cells, the cells were divided into the control group and LPS group (1ug/ul). Then, the astrocytes/BV2 cells were treated with mimic-miR-122-5p or mimic-nc, then divided into four groups: control group, LPS group, mimic-nc+LPS group and mimic miR-122-5p+LPS group (**Supplementary Figure 2A**).

#### 2.10.2 Group II

To clarify the effect of lncRNA xist on inflammatory cytokines release, purified astrocytes/BV2 cells were cultured and treated with si-lncRNA xist, then divided into four groups: control group, LPS group, si-lncRNA nc+LPS group and si-lncRNA xist+LPS group (**Supplementary Figure 2B**).

#### 2.10.3 Group III

To investigate whether miR-122-5p regulates inflammatory cytokines generation through downstream target gene PKCη to activate NF-κB signaling pathway, purified astrocytes/BV2 cells were administrated with si-PKCη, then divided into four groups: control group, LPS group, si-nc+LPS group and si-PKCη+LPS group (**Supplementary Figure 2C**).

### 2.11 293T cells cultures

The 293T cells were obtained from the Public Laboratory of Guangdong Provincial People’s Hospital, and the certificate number for 293T cells is No. PC-H2020082502. Cells were routinely maintained in DMEM supplemented with 10% fetal bovine serum (FBS) at 37°C in a humidified atmosphere with 5% CO2. To verify the targeted relationship between miR-122-5p and PKCη, 293T cells were cultured, then co-transfected with mimic miR-122-5p or mimic-nc, or a plasmid with the wild-type or mut-type PRKCH 3′-UTR. They were divided into four groups: mimic nc+wild-type PKCη, mimic nc+mut-type PKCη, mimic miR-122-5p+wild-type PKCη, mimic miR-122-5p+mut-type PKCη (**Supplementary Figure 2D**).

### 2.12 Cell viability assays

To exclude the effect of transfection reagents on the activity of astrocytes/BV2 cells, cell viability was analyzed using the Cell Counting Kit-8. Cells (1000 cells/well) were seeded into a 96‐well plate and treated with transfection reagent for 6 hours. Cell viability was measured at an absorbance at 450 nm using a microplate reader.

### 2.13 Cell transfection

siRNAs (mimics miR-122-5p, mimics nc, si-lncRNA xist and si-lncRNA nc) and plasmids (wild-type/mut-type PKCη) were purchased from Ribo (Guangzhou, China). Transfection were performed when astrocytes/BV2 cells density had risen to 70%. Transfection methods were used according to the instructions. At 24h after transfection, astrocytes/BV2 cells were administrated with LPS for 6h. The transfection efficiency was detected by qRT-PCR. The plasmids containing wild-type or mut-type PKCη were co-transfected with miR-122-5p mimics or nc into 293T cells using Lipofectamine 3000 (Invitrogen) according to the manufacturer’s protocols.

### 2.14 Real-time quantitative PCR

Total RNAs were isolated from tissues or primary astrocytes or BV2 cells using Trizol reagent (Invitrogen). RNAs were transcribed into cDNA using Prime-Script RT Reagent Kit (Takara, Dalian, China). qPCR was performed using SYBR Prime Script RT-PCR kits (AG) on ABI 7300 Fast Real-Time PCR system (Applied Biosystems, Foster City, CA) according to the manufacturer’s instructions. U6 was used as the normalization control for miRNA in cell and tissue, while GAPDH was used as the normalization control for other genes. Relative expression was calculated using the 2^-ΔΔCt^ method.

miRNA was extracted from cerebrospinal fluid as follows: Fresh cerebrospinal fluid was centrifuged at 2000rpm at 4°C for 5min. After centrifugation, the clear cerebrospinal fluid was carefully aspirated into a clean EP tube. Subsequently, 5ul cel-miR-39-3p was added as an external reference to each sample, which was followed by adding 750μl Trizol for lysis. After centrifugation, the supernatant was transfered to a new labeled enzyme-free EP tube, and 150ul chloroform were added. The EP tube was thoroughly mixed by vortex oscillation and placed on ice for 10 minutes. Then, samples were centrifuged at 12000 rpm/min for 15 minutes at 4°C. The upper aqueous phase was transfered into a new 1.5ml EP tube (approximately 1ml), which was placed at room temperature for 5 minutes to fully lyse the tissue cells. After centrifugation, the sample will be separated into three layers. The RNA was carefully extracted from the aqueous phase using an enzyme-free pipette tip and an equal volume of isopropanol was added. The sample was thoroughly mixed by vortex oscillation and placed on ice for 10 minutes, then centrifuged at 12000 rpm/min for 15 minutes at 4°C. The supernatant was discarded, and the bottom precipitate was RNA. 75% cold ethanol was prepared in advance and used to wash away organic solvents around the RNA. The sample was centrifuged at 12000 rpm/min for 5 minutes at 4°C. The supernatant was discarded and the EP tube upside down was placed on a drying rack for 10 minutes. The RNA was dissolved with nuclease-free water, and the RNA concentration was measured.

### 2.15 Double immunofluorescence

Rat brain tissues in this study were fixed in 4% paraformaldehyde, then followed by dehydration with 20% and 30% sucrose. Frozen sections were cut at 10μm thickness, then mounted on glass slides and stored in a cryoprotectant at -20. Coronal brain sections derived from different groups were divided into three groups. The brain sections in *Group I*, which were obtained from the PWM of rats sacrificed at 6h, 24 h, 3 and 7 days after CLP, as well as corresponding controls, and incubated with antibody directed against anti-IL-1β and anti-Iba1 (a microglia marker) or anti-GFAP (an astrocyte marker). The brain sections in *Group II* and *Group III* were from the PWM of rats sacrificed at 24 h after CLP + ICV-AAV-miR-122-5p or CLP+ ICV-ASO-lncRNA xist treatment, and were incubated with antibody directed against anti-IL-1β and anti-Iba1or anti-GFAP. Primary antibodies were incubated overnight at 4°C. After washing three times by PBS, the sections were incubated at room temperature for 1h with appropriate fluorescent secondary antibodies: goat anti-chicken IgG (1:300, Alexa Fluor 488, Abcam, ab150169), goat anti-rabbit IgG (1:300, Alexa Fluor 488, Abcam, ab150077), goat anti-mouse IgG (1:300, Alexa Fluor 594, Abcam, ab150116), goat anti-rabbit IgG (1:300, Alexa Fluor 594, Abcam, ab150080). After three washes with PBS, DAPI (Sigma-Aldrich, D9542) was used for counterstaining of the nucleus.

Quantitative analysis of the cell number of IL-1β-positive microglial cells or astrocytes in the PWM of rats in different groups (n = 3) was carried out through counting labeled cells in eight randomly selected microscopic fields from sections obtained from three rats at x40 objective, respectively. The cells with a green Iba1 or GFAP labeling fluorescent cell body overlapping with red were counted as IL-1β positive cells, while those cell bodies emitting only green fluorescence were counted as IL-1β-negative cells. The positive cell count was performed using Image J software.

BV2 cells were treated with LPS or mimic nc + LPS or mimic miR-122-5p + LPS. After treatment, cells were fixed in 4% PFA for 30 minutes, then incubated with 0.3% Triton X-100 (Aladdin, T109027) in PBS for 15 minutes and blocked with 10% normal goat serum in 0.3% Triton X-100 at room temperature for 1h. Then, the BV2 were incubated overnight with primary antibodies anti-Iba1 and anti-IL-1β antibody at 4°C. On the following day, the cells were washed for three times and incubated with goat anti-mouse IgG (1:200, Alexa Fluor 594, Abcam, ab150116) or goat anti-rabbit IgG (1:200, Alexa Fluor 594, Abcam, ab150080) for 1 h. After a final washing step with PBS, the cells were mounted on glass slides and contained DAPI.

The primary astrocytes were treated with LPS or mimic nc + LPS or mimic miR-122-5p + LPS. After treatment, the cells were fixed in 4% PFA for 30 minutes, then incubated with 0.3% Triton X-100 (Aladdin, T109027) in PBS for 15 minutes and blocked with 10% normal goat serum in 0.3% Triton X-100 at room temperature for 1h. Then, the primary astrocytes were incubated with anti-GFAP and anti-IL-1β antibody overnight at 4°C. On the following day, the cells were washed and incubated with goat anti-mouse IgG (1:200, Alexa Fluor 594, Abcam, ab150116) or goat anti-rabbit IgG (1:200, Alexa Fluor 594, Abcam, ab150080) for 1 h. After a final washing step with PBS, the cells were mounted on glass slides and contained DAPI.

### 2.16 FISH combination with immunostaining

The cultured dishes of primary astrocytes or BV2 cells were fixed in 4% PFA for 10 minutes and washed with PBS for three times. The cultured spheres were permeabilized with 0.3% Triton X-100 in PBS for 15 minutes and prehybridized in hybridization buffer for 1 h at 37°C. Hybridization buffer containing with 50 nM commercially available biotin-labeled lncRNA xist probe was heated to 65°C for 5 minutes and added to each position of the slide, which were then allowed to hybridize at 37°C overnight. The next day, the sections were washed for 3 times in 2x SSC and twice in 0.2x SSC at 42°C. The sections were blocked with 1% BSA and 3% normal goat serum in PBS for 1 h at room temperature and then incubated with anti-GFAP or Iba1 antibody overnight at 4°C. On the third day, after the cells were washed for 3 times with TBS, they were incubated with goat anti-mouse IgG (1:200, Alexa Fluor 594, Abcam, ab150116) for 1 h at room temperature. The samples were washed for three times in PBS and were then incubated with DAPI to visualize the nuclei. Immunofluorescence images were captured using confocal microscopy.

### 2.17 Enzyme-linked immunosorbent assays

The levels of IL-1β and TNF-α in the CSF of ICI and non-ICI patients were measured using ELISA kits according to the manufacturer’s protocol. Firstly, collected CSF samples were centrifuged. Secondly, 100ul of the supernatants were aspirated. Then, the levels of IL-1β (CUSABIO, CSB-E08053h, USA) and TNF-α (CUSABIO, CSB-E04740h, USA) in supernatants were measured by ELISA kits, respectively. Analysis of optical density was performed in a multifunctional microplate reader.

### 2.18 Western blotting

The proteins from the PWM of the rats or primary astrocytes or BV2 cells with different treatments were extracted using a protein extraction kit (Best Bio, BB-3101-100T). The standard protocol as previously described (26) was used to detect the protein concentrations by BCA Protein Assay Kit (Thermo Scientific, 23250). Samples of supernatants containing 30 µg of total protein were heated at 100°C for 10 min. Samples were separated by SDS-PAGE. Following electrophoresis, the samples were transferred onto 0.22 μm polyvinylidene fluoride membranes (Bio-Rad, USA). The membranes were blocked in 5% nonfat dried milk in Tris-buffered saline containing 0.05% Tween 20 (TBST) for 1 h at room temperature, then incubated with primary antibodies according to the manufacturer’s instructions. The primary antibodies and dilution concentration used in western blot are listed in **Supplementary Table S1**. After three washes in TBST, the membranes were incubated with the appropriate secondary antibodies: anti-mouse IgG (1:3000, Cell Signaling Technology, 7076S) or anti-rabbit IgG (1:3000, Cell Signaling Technology, 7074S) for 1h at room temperature. The protein bands were visualized by chemiluminescence kit (Millipore, WBKLS0500) and images were created by Image Quant LAS 500 Imager (GE Healthcare Bio-Sciences AB). The optical density of the respective protein bands was quantified with image J software.

### 2.19 Dual-luciferase reporter gene assay

Dual-Luciferase Reporter Gene Assay Target genes of miRNA-122-5p were predicted using TargetScan (http://www.targetscan.org/vert_71/). The binding sites between PKCη and miR-122-5p were predicted. Plasmids containing wild-type (WT) PKCη Luciferase reporter gene vector and mutant (Mut) PKCη Luciferase reporter gene vector were constructed. They were co-transfected with miR-122-5p mimics or negative control (NC), into 293T cells respectively. At 24h after transfection, the luciferase activity was determined by a Dual-luciferase Reporter Assay System (Promega) according to the manufacturer’s instructions. For the targeting relationship between lncRNA xist and miR-122-5p, literatures have already proved (27).

### 2.20 Statistical analysis

**Figure 1:**
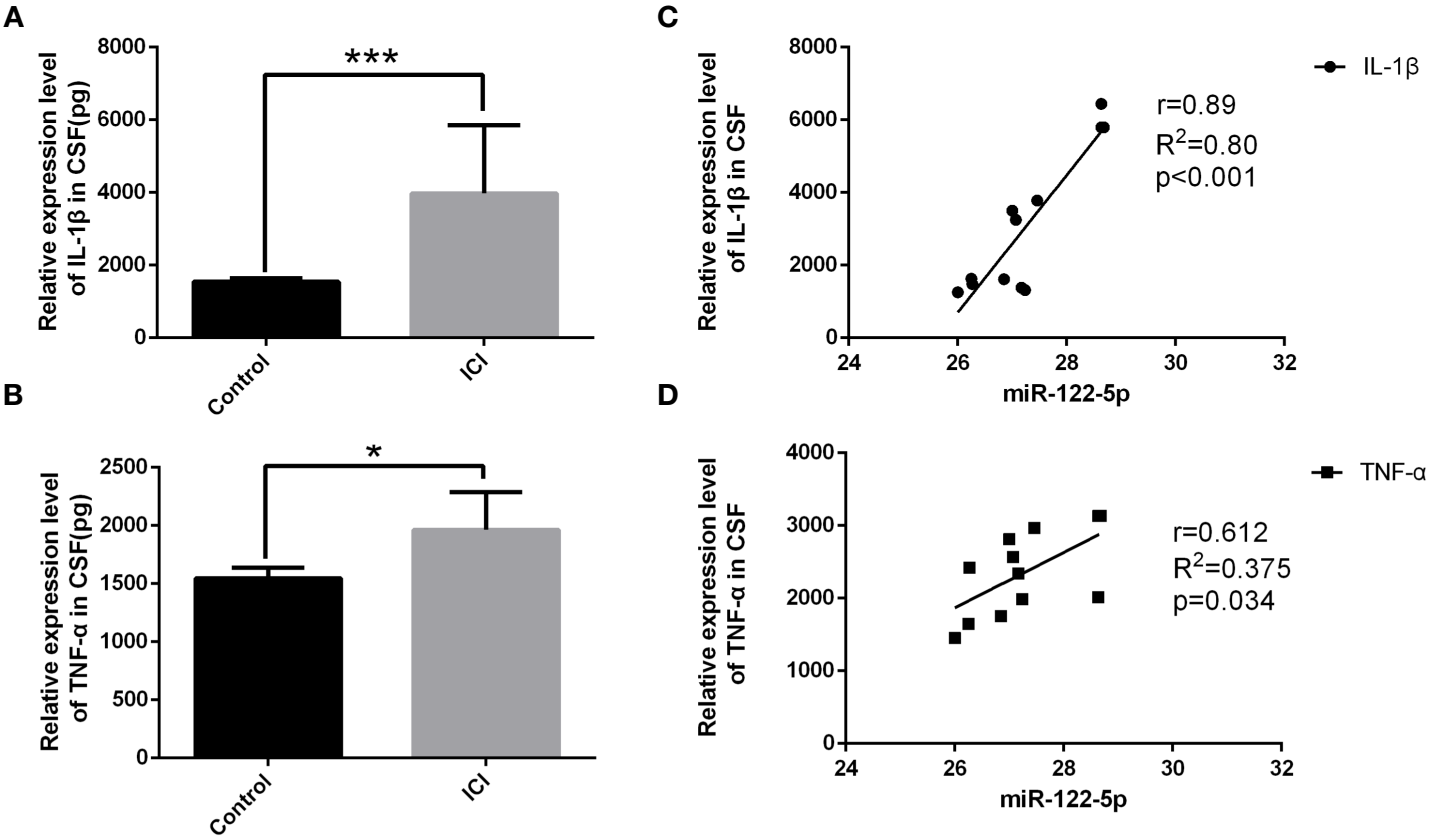
The expression of miR-122-5p was downregulated in the CSF of ICI patients. Quantification by ELISA shows significant increase in the cytokine secretion of IL-1β **(A)** and TNF-α **(B)** in the CSF in ICI patients when compared with the non-ICI patients. The associations of miR-122-5p relative expression with IL-1β and TNF-α in ICI patients were analyzed. miR-122-5p was negatively correlated with IL-1β **(C)** and TNF-α **(D)** in ICI patients. miR-122-5p relative expression between non-ICI patients and ICI patients were assessed by Wilcoxon rank-sum test. The associations of miR-122-5p expression with IL-1β and TNF-α in ICI patients were evaluated by *Pearson* correlation test. *p<0.05 and ***p< 0.001 were considered significant. n=4 for non-ICI group, n=12 for ICI group.

**Figure 2:**
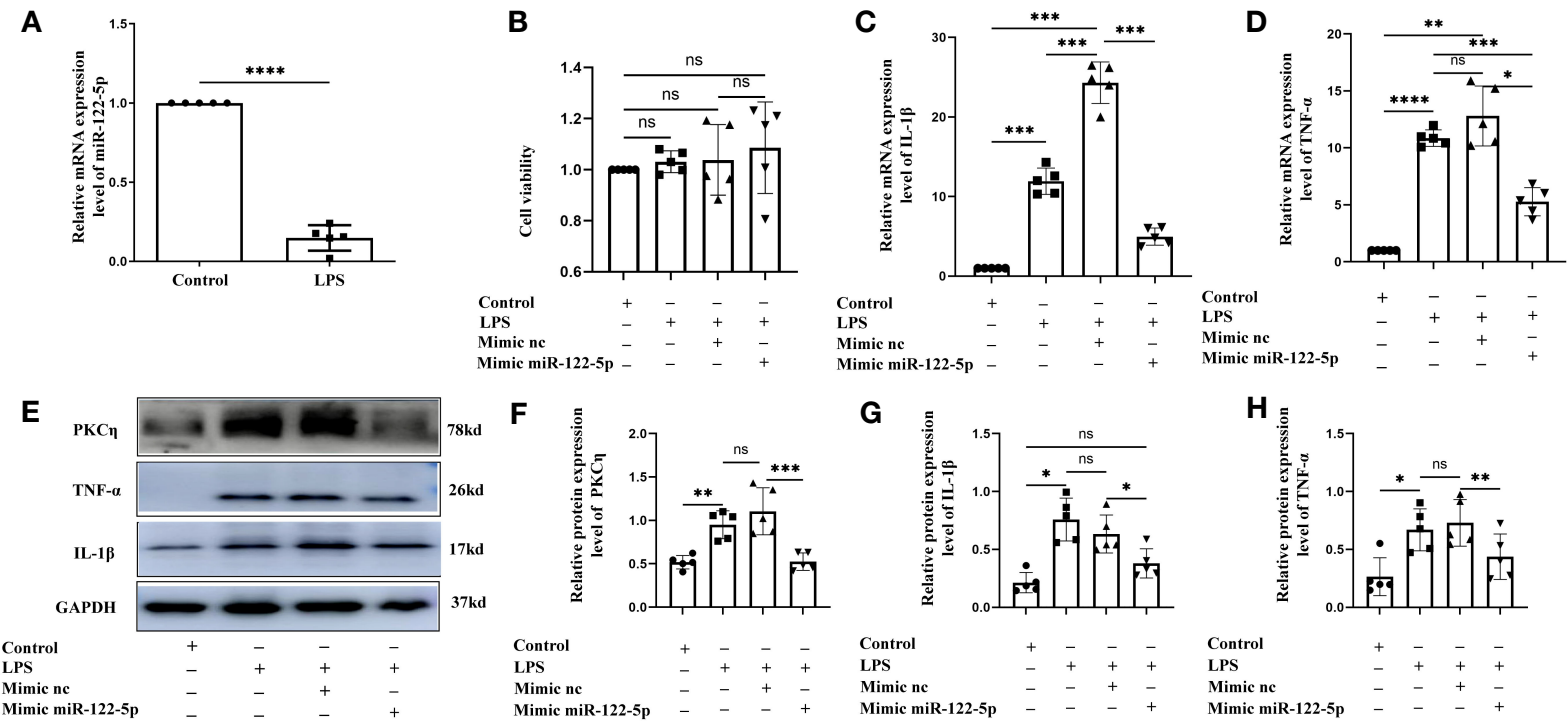
Mimic miR-122-5p could reduce the production of IL-1β and TNF-α in BV2 cells treated with LPS *in vitro*. **(A)** miR-122-5p expression in the BV2 cells after LPS stimulation. **(B)** CCK8 assay tested the effect of transfection reagents on the viability of BV2 cells. **(C, D)** mRNA expression of IL-1β **(C)** and TNF-α **(D)** in the BV2 cells after LPS stimulation, mimic-nc+LPS treatment or mimic-miR-122-5p+LPS treatment were detected by RT-PCR. **(E)** shows the immunoreactive bands of PKCη (78kDa), IL-1β (17kDa), TNF-α (26kDa) and GAPDH (37kDa) after LPS stimulation, mimic-nc+LPS treatment, mimic-miR-122-5p+LPS treatment, and the corresponding control (n=5 for each group). Bar graphs **(F–H)** show the optical density of protein expression shown in Panel E (n=5 for each group). For statistical analysis, Student t tests was used for **(A)** and one-way ANOVA followed by Holm-Sidak tests was used for **(B–D), (F–H)** and presented as the mean ± standard error of measurement (SEM). *p<0.05, ** *p <*0.01, *** *p <*0.001.

**Figure 3:**
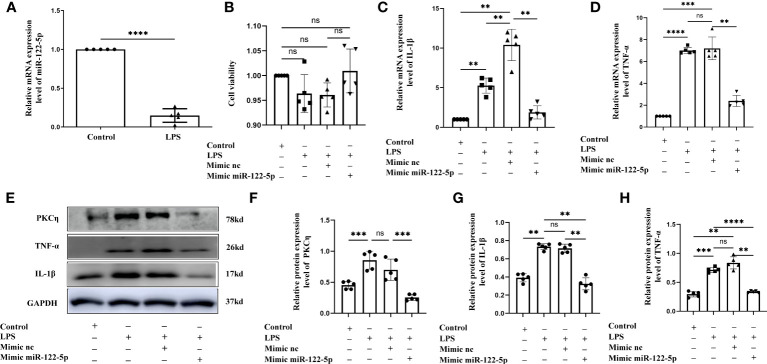
Mimic miR-122-5p could reduce the production of IL-1β and TNF-α in astrocytes treated with LPS *in vitro*. **(A)** miR-122-5p expression in the astrocytes after LPS stimulation. **(B)** CCK8 assay tested the effect of transfection reagents on the viability of astrocytes. **(C, D)** mRNA expression of IL-1β **(C)** and TNF-α **(D)** in astrocytes after treatment with LPS, mimic-nc+LPS or mimic-miR-122-5p+LPS were detected by RT-PCR. **(E)** shows the immunoreactive bands of PKCη (78kDa), IL-1β (17kDa), TNF-α (26kDa) and GAPDH (37kDa) after LPS, mimic-nc+LPS, mimic-miR-122-5p+LPS, and the corresponding control (n=5 for each group). Bar graphs **(F–H)** show the optical density of protein expression in **(E)** (n=5 for each group). For statistical analysis, student t tests was used for **(A)** and one-way ANOVA followed by Holm-Sidak tests was used for **(B–D)**, **(F–H)** and presented as the mean ± standard error of measurement (SEM). *p<0.05, ** *p <*0.01, *** *p <*0.001.

**Figure 4:**
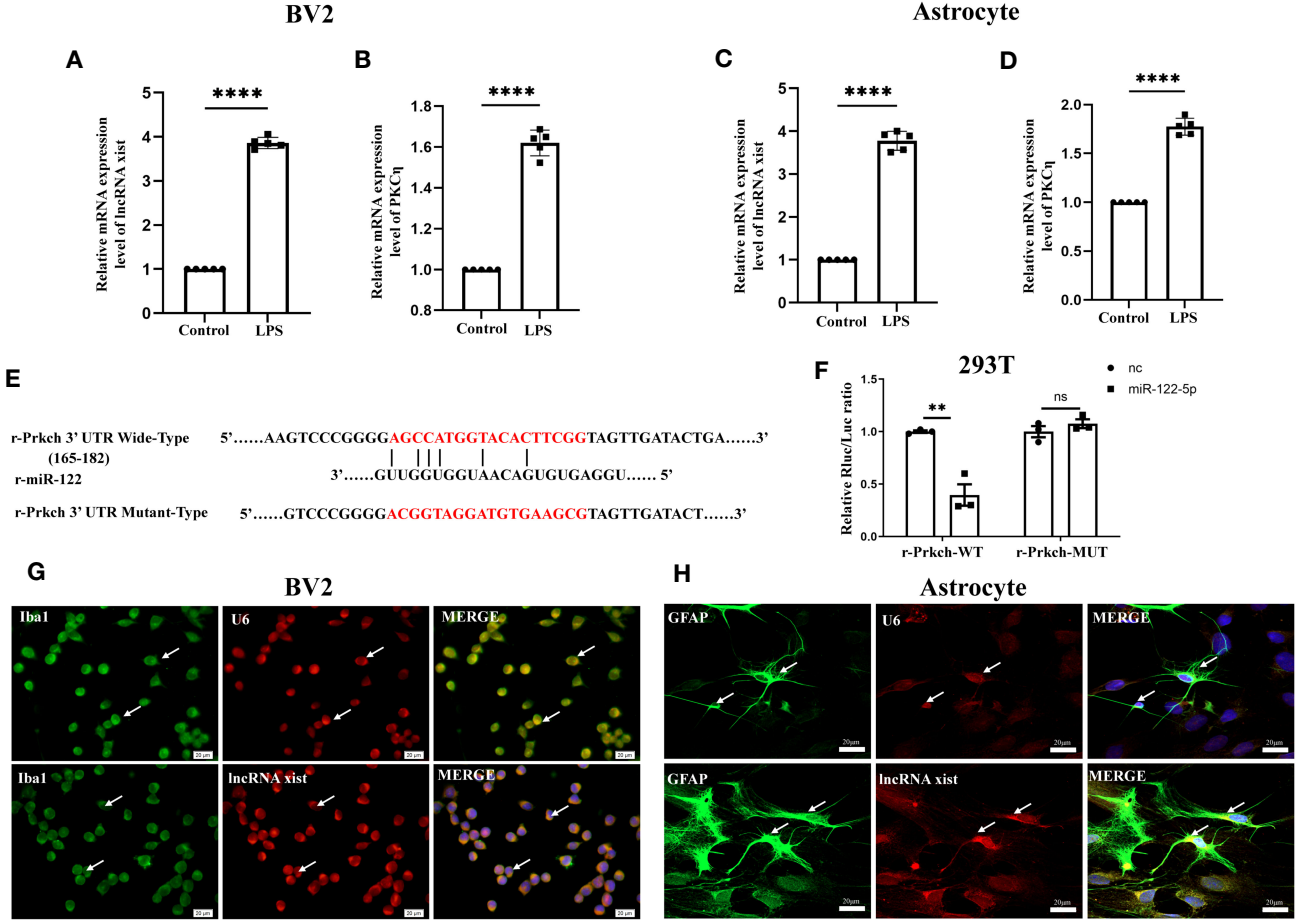
Upstream and downstream target genes of miR-122-5p. **(A, B)** The mRNA expression of lncRNA xist **(A)** and PKCη **(B)** in BV2 cells were detected by qPCR (n=5 for each group). **(C, D)** The mRNA expression of lncRNA xist **(C)** and PKCη **(D)** in astrocytes were detected by qPCR (n=5 for each group). **(E)** Predicted miR-122-5p target sequence in PKCη-3′ UTRs. Target sequences of PKCη-3′ UTRs were mutated. **(F)** Luciferase assay was detected in 293T cells transfected with PKCη-3′ UTR-WT or PKCη-3′ UTR-MUT reporter together with miR-122-5p or miR-nc mimic (n=3 for each group). **(G, H)** FISH assay showed that lncRNA xist was expressed in the cytoplasm of BV2 cells and astrocytes. For statistical analysis, Student t tests was used for **(A–D, F)** and presented as the mean ± standard error of measurement (SEM). U6, positive control. Scale bars: G, H, 20µm. *p<0.05, ** *p <*0.01, *** *p <*0.001.

**Figure 5:**
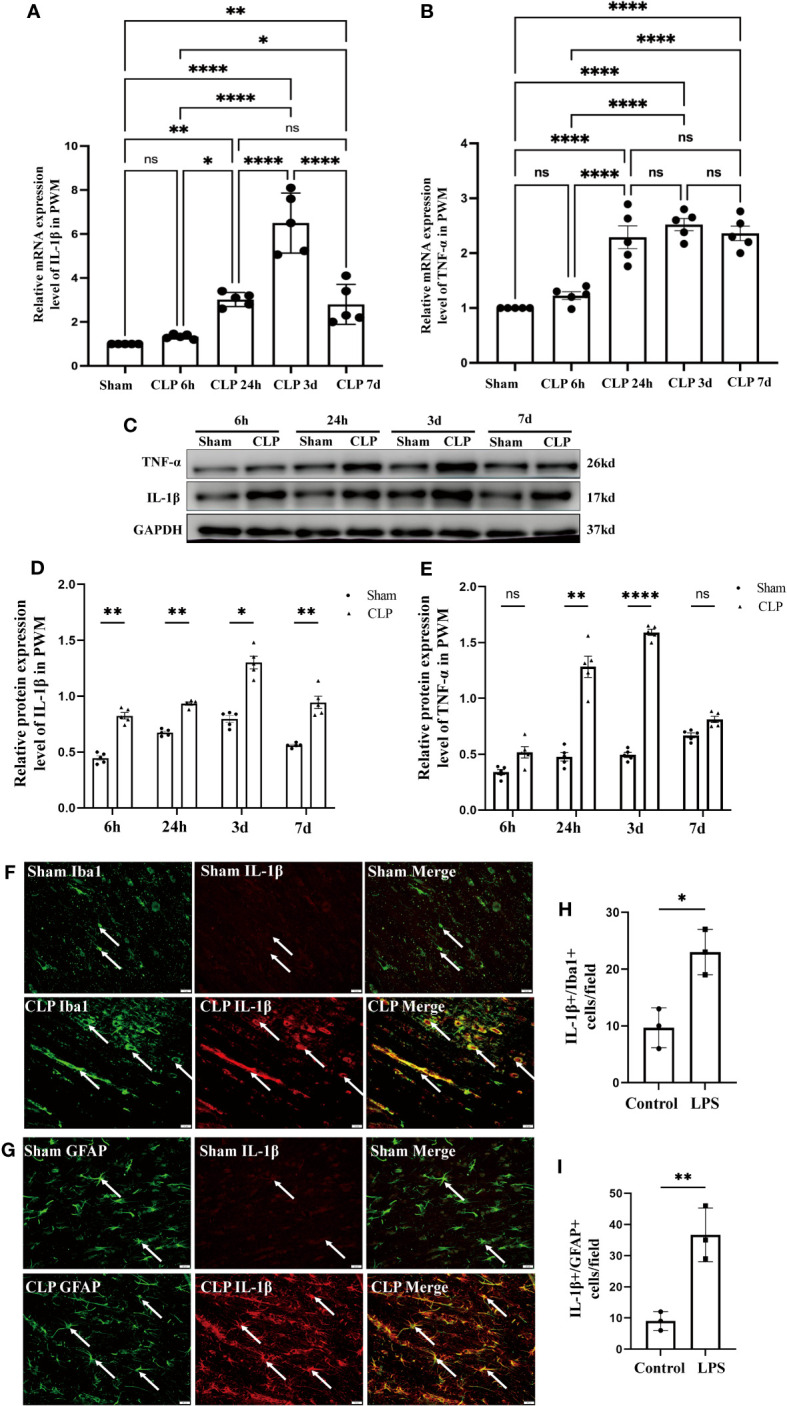
Expression of IL-1β and TNF-α in the PWM of CLP septic rats. mRNA expression of IL-1β **(A)** and TNF-α **(B)** in the PWM at 6h, 24h, 3d, and 7d after CLP were detected by RT-PCR (n=5 for each group). **(C)** shows the immunoreactive bands of IL-1β (17kDa) and TNF-α (26kDa) at 6h, 24h, 3d, and 7d after CLP and the sham group. Bar graphs **(D, E)** show the optical density of protein expression in Panel **C** (n=5 for each group). Double immunofluorescence staining shows the distribution of Iba1 (**F**, green), GFAP (**G**, green) and IL-1β (**F, G**, red) immunoreactive microglial cells or astrocytes in the PWM at 24 h after CLP operation and their matching controls. Co-localized expression of Iba1 or GFAP and IL-1β could be seen in **(F, G)** The bar graph in H and I show a significant increase in the number of IL-1β^+^/Iba1^+^ or IL-1β^+^/GFAP^+^ cells in the PWM at 24 hours after CLP in comparison with their corresponding controls. Note IL-1β expression in microglia or astrocytes was increased in the PWM at 24 h after CLP operation when compared with control. For statistical analysis, one-way-ANOVA followed by Holm-Sidak tests was used for **(A, B)** and presented as the mean ± standard error of measurement (SEM). PWM, periventricular white matter; CLP, cecal ligation perforation. *p<0.05, **p <0.01, ***p <0.001 Scale bars: F, 20µm.

**Figure 6:**
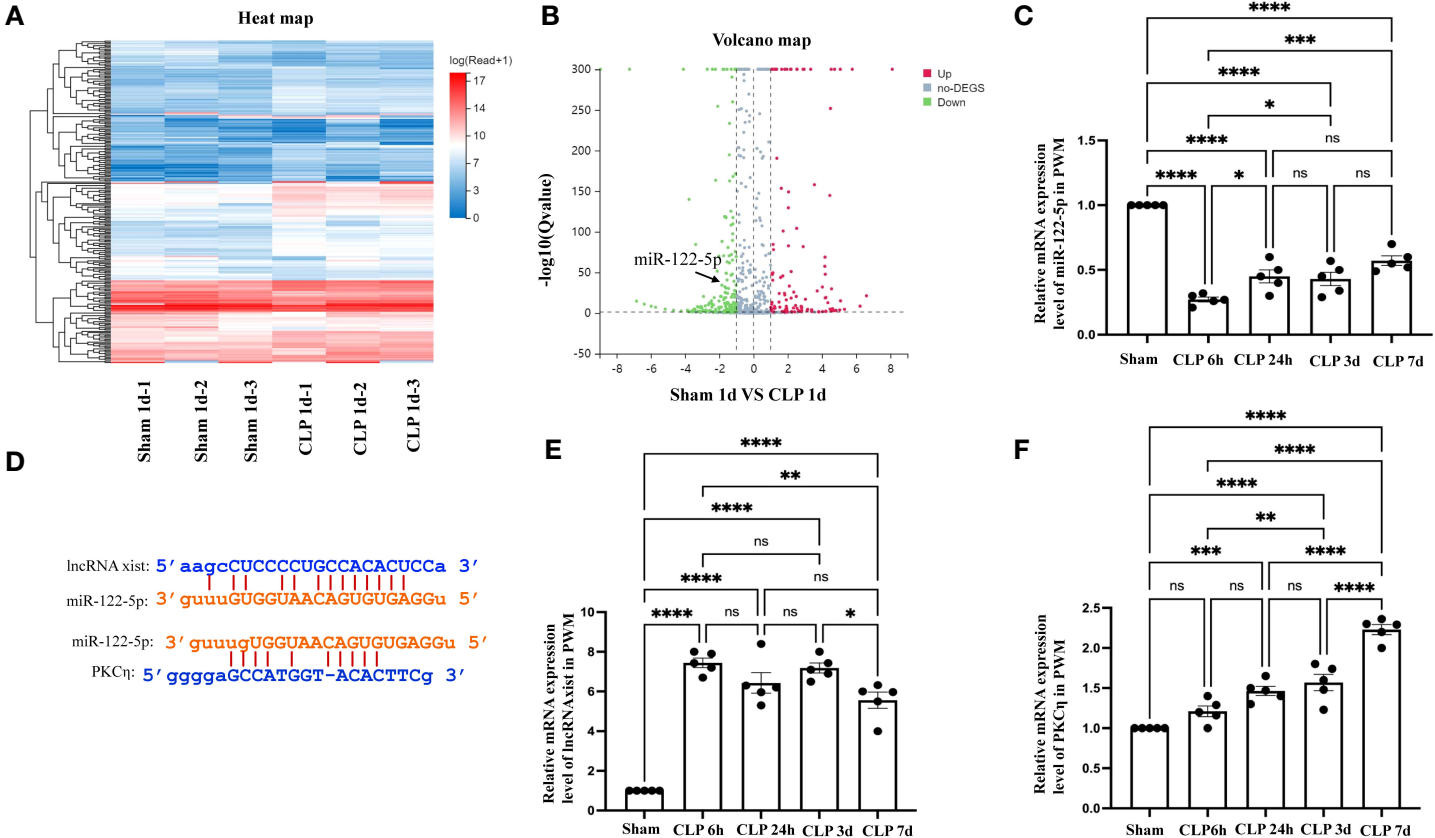
The level of miR-122-5p was downregulated in the PWM of CLP rats. **(A)** The hierarchical clustering of partial differentially expressed miRNA in the PWM of sham and CLP septic rats. ‘Red’ indicates high relative expression, and ‘blue’ indicates low relative expression. **(B)** Scatter Plot of miRNAs expression levels in the PWM. ‘Red’ represents up-regulation and ‘blue’ represents down-regulation more than 1.5-fold change of miRNAs between sham group and CLP group. miR-122-5p is indicated by black arrows. **(C)** miR-122-5p expression in the PWM at 6h, 24h, 3d, and 7d after CLP was detected by RT-PCR (n =5 per group). **(D)** Upstream and downstream target genes of miR-122-5p were lncRNA xist and PKCη, respectively, which was predicted using TargetScan and miRbase. **(E, F)**. lncRNA xist and PKCη expression in the PWM at 6h, 24h, 3d, and 7d after CLP were detected by RT-PCR (n =5 per group). For statistical analysis, one-way-ANOVA followed by Holm-Sidak tests was used for **(C, E, F)** and presented as the mean ± standard error of measurement (SEM). PWM, periventricular white matter; CLP, cecal ligation perforation. *p<0.05, ** *p <*0.01, *** *p <*0.001.

**Figure 7:**
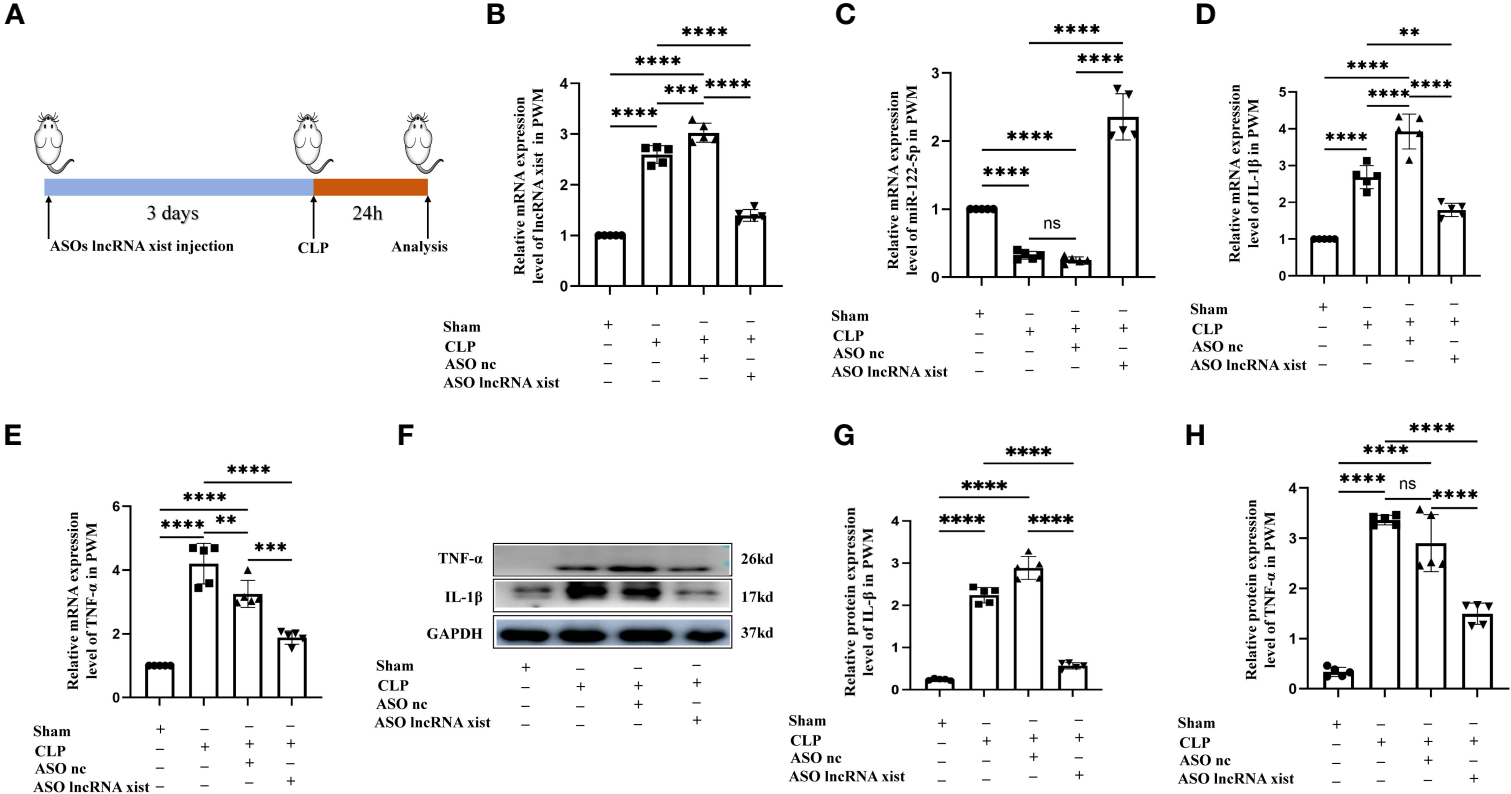
Knockdown of lncRNA xist expression inhibits neuroinflammation in the PWM of CLP septic rats. **(A)** The animal flow chart of knockdown lncRNA xist. **(B, C)** lncRNA xist **(B)** and miR-122-5p **(C)** expression in the PWM of rats at 24h after CLP, CLP+ASO lncRNA xist, CLP+ASO lncRNA nc or sham as detected by RT-PCR (n=5 for each group). **(D, E)**. mRNA expression of IL-1β **(D)** and TNF-α **(E)** in the PWM of rats at 24h after CLP, CLP+ASO lncRNA xist, CLP+ASO lncRNA nc or sham as detected by RT-PCR. **(F)** shows the immunoreactive bands of IL-1β (17kDa), TNF-α (26kDa) and GAPDH (37kDa) at 24h after CLP, ASO lncRNA-nc +CLP, ASO lncRNA-xist +CLP treatment and sham group (n=5 for each group). Bar graphs **(G, H)** show the optical density of protein expression in **(F)** (n=5 for each group). For statistical analysis, one-way ANOVA followed by Holm-Sidak tests was used for **(B–E, G–H)** and presented as the mean ± standard error of measurement (SEM). PWM, periventricular white matter; CLP, cecal ligation perforation. ASO, antisense oligonucleotide. *p<0.05, ** *p <*0.01, *** *p <*0.001.

**Figure 8:**
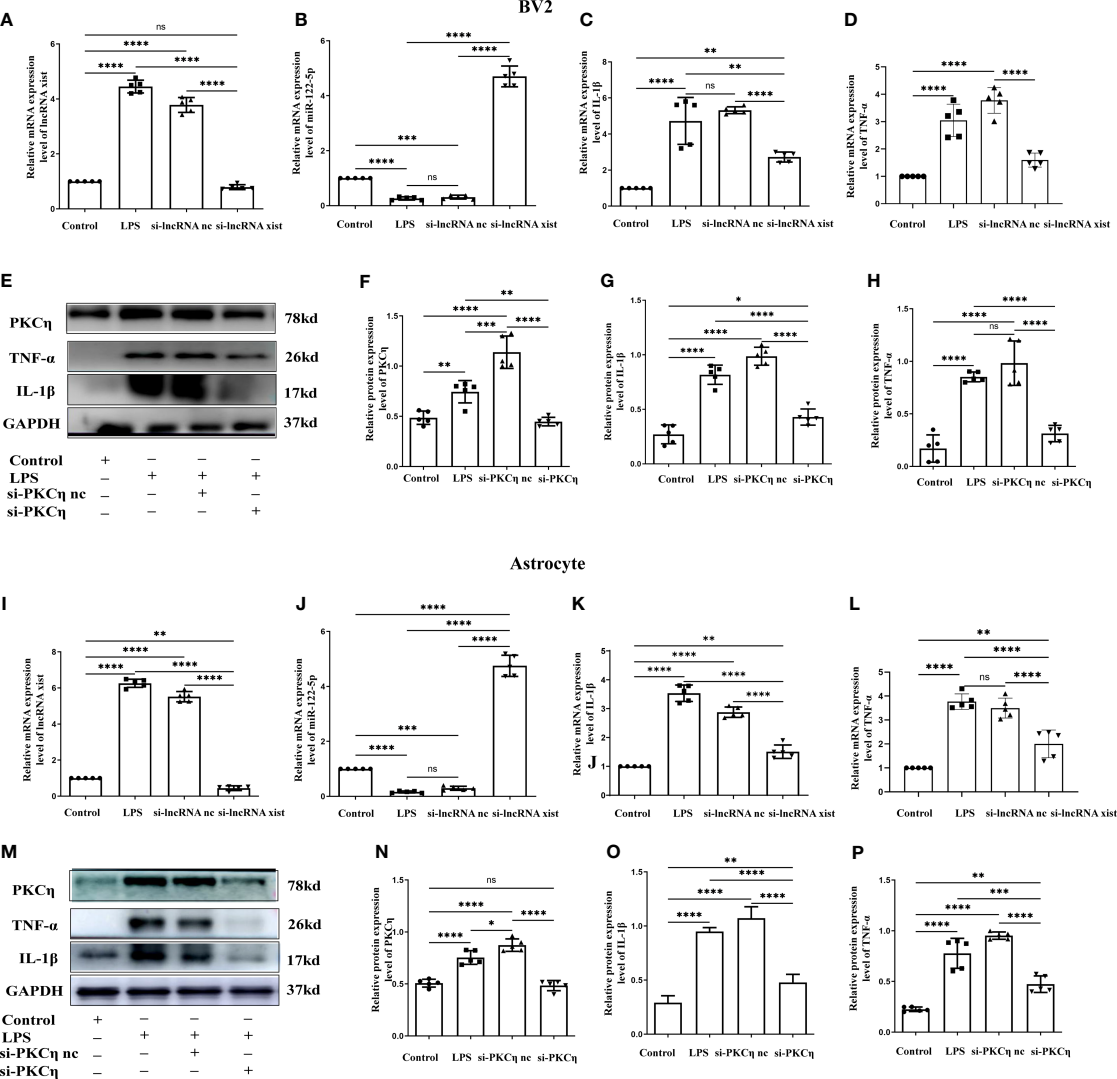
Knockdown of lncRNA xist and PKCη attenuated the generation of IL-1β and TNF-α in BV2 cells and astrocytes administrated with LPS. The mRNA level of lncRNA xist **(A, I)** and miR-122-5p **(B, J)** in BV2 cells or astrocytes after LPS, si-lncRNA nc +LPS or si-lncRNA xist+LPS were detected by RT-PCR. mRNA expression of IL-1β **(C, K)** and TNF-α **(D, L)** in BV2 cells or astrocytes after LPS, si-lncRNA nc +LPS or si-lncRNA xist+LPS were detected by RT-PCR. **(E, M)** in BV2cells and astrocytes showed the immunoreactive bands of PKCη (78kDa), IL-1β (17kDa), TNF-α (26kDa) and GAPDH (37kDa) after LPS, si-PKCη-nc+LPS, si-PKCη+LPS, and the corresponding control (n=5 for each group). Bar graphs **(F–H)** show the optical density of protein expression in **(E)** (n=5 for each group). Bar graphs **(N–P)** show the optical density of protein expression in Panel M (n=5 for each group). For statistical analysis, one-way ANOVA followed by Holm-Sidak tests was used and all data were presented as the mean ± standard error of measurement (SEM). *p<0.05, *p<0.05, ** *p <*0.01, *** *p <*0.001.

Statistical analysis was performed using GraphPad Prism 9.2.0 Software. All data are presented as the mean ± SEM. Different statistical methods were used to analyze different types of data. The data in **Figures 1C, D** were analyzed using Pearson correlation analysis. The data in **Figures 2A**, **3A**, **4**, **5D, E, H, I** were analyzed using Student t tests. The data in **Figures 2B–G**, **3B–G**, **6**–**8**, and **Supplementary Figures 3–8** were analyzed using one-way ANOVA followed by Holm-Sidak tests. The appropriate test is showed in the figure legends. The results were judged to be statistical significance if *p*<0.05 by analysis of variance.

## 3 Results

### 3.1 The expression of miR-122-5p was downregulated in the CSF of ICI patients

To determine whether miRNAs were involved in neuroinflammation, the levels of miR-122-5p were detected in the CSF of ICI and non-ICI patients. According to the inclusion and exclusion criteria, 4 non-ICI and 12 ICI patients were included in this study. Demographic characteristics included age, gender, ICU stay time, ventilator duration, APACHE II, SOFA, GCS, Nervous system positive signs, seizures, MODS. CSF samples were collected from ICI patients [age 55 (53.75, 73.75) years] and non-ICI patients [age 44 (28, 55.75) years]. ICI patients had longer ICU stay time when compared with non-ICI patients. The sociodemographic and clinical characteristics were listed in **Table 1**. Biochemical indexes included white blood cell (WBC), procalcitonin (PCT), C-reactive protein (CRP), serum creatinine (Scr), albumin (Alb), bilirubin (BIL), sputum culture, urine culture, cerebrospinal fluid routine and cerebrospinal fluid biochemistry. The WBC, Pandy’s test, and microalbumin in CSF of ICI patients were higher than these in the non-ICI patients. The laboratory parameters of these patients were shown in **Table 2**. The Cycle threshold (Ct) levels of miR-122-5p in non-ICI group (n=4) and ICI group (n=12) are 13.9 ± 2.29 and 19.7 ± 4.06, respectively. The level of miR-122-5p were significantly decreased (*p*= 0.004) in the CSF of ICI patients when compared with non-ICI group. The levels of tumor necrosis factor-α (TNF-α) and interleukin-1β (IL-1β) in the CSF were measured by ELISA. The levels of IL-1β or TNF-α in the CSF of ICI patients were higher than these in the non-ICI group (**Figures 1A, B**). Pearson correlation analysis was performed and showed that there were negative correlations between miR-122-5p and IL-1β (**Figure 1C**) or TNF-α (**Figure 1D**).

### 3.2 The level of lncRNA xist, miR-122-5p and PKCη in the PWM of CLP septic rats

To investigate the potential involvement of miRNAs in the occurrence of SAN, we performed a high-throughput sequencing analysis of miRNAs level in the PWM of CLP 1d rats and corresponding control. 2069 miRNAs were obtained through this sequencing analysis. A fold change threshold of 1.5 and a p-value <0.05 were also set to produce a separate dataset. We here reported that there were 93 high differential expression miRNAs and 145 low differential expression miRNAs. The sequence read archive of high-throughput sequencing were uploaded to the NCBI and the submission number is SUB13472312.The heatmap and Scatter-Plot were performed to miRNAs according to their different expression levels (**Figures 6A, B**). Among these miRNAs, miR-122-5p expression was downregulated with 1.5-fold changes in CLP group. Then, the miR-122-5p expression was validated through qPCR in the PWM of rats in sham group and CLP group at 6h, 24h, 3d, and 7d after CLP operation. The results showed that miR-122-5p expression in the CLP group was saliently decreased at 6h-7d after CLP operation, and the level of miR-122-5p was at low level with no significant difference at 24-7d after CLP operation (**Figure 6C**). Subsequently, the upstream and downstream target genes of miR-122-5p were predicted by bioinformatics. We reported that lncRNA xist and PKCη were the upstream and downstream target genes of miR-122-5p, respectively (**Figure 6D**). The levels of lncRNA xist and PKCη mRNA in the PWM of CLP rats were also detected by qPCR. The results showed that the mRNA expression of lncRNA xist in the CLP group was significantly increased at 6h-7d after CLP operation (**Figure 6E**). The mRNA level of PKCη in the CLP group was significantly upregulated at 24h, 3d and 7d after CLP operation (**Figure 6F**). However, there was no significant difference at 6h (**Figure 6F**).

### 3.3 Expression of IL-1β and TNF-α in the PWM of CLP septic rats

To explore the neuroinflammation in the PWM of CLP septic rats, we first examined the mRNA and protein expression of proinflammatory mediators such as IL-1β and TNF-α. The mRNA levels of IL-1β and TNF-α in the PWM were significantly upregulated at 24h, 3d and 7d after CLP, peaking at 3d, when compared with the corresponding controls (**Figures 5A, B**). However, no significant difference was found at 6h as compared with the sham group (**Figures 5A, B**). In order to exclude the impact of surgical procedures, we also performed time-points intervention in the sham group in the western blot test. The optical density of immunoreactive bands of IL-1β protein which appeared at approximately 17kDa was markedly enhanced at 6 and 24 h, 3 and 7 days in CLP group, peaking at 3d when compared with the corresponding controls (**Figures 5C, D**). Upregulation of TNF-α protein that appeared at approximately 26kDa was markedly enhanced at 24 h and 3 days in CLP, peaking at 3d, as compared with the corresponding controls (**Figures 5C, E**). There were no significant differences between the protein level of TNF-α at 6h and 7d when compared with the corresponding controls (**Figures 5C, E**). Furthermore, double immunofluorescence showed that IL-1β antibody and Iba1 were colocalized in activated microglia in the PWM of CLP septic rats (**Figure 5F**). At 24h following CLP operation, immunoreactivity for IL-1β was enhanced in large numbers of Iba1^+^ microglia (**Figures 5F, H**). Similarly, double immunofluorescence also showed an increase in the number of IL-1β^+^/GFAP^+^ astrocytes in the PWM of rats at 24 h after CLP when compared with the sham group (**Figures 5G, I**). Taken together, these results demonstrated that microglia and astrocytes were activated and produced proinflammatory mediators in the PWM of CLP septic rats. In other words, neuroinflammation occurred in the PWM of CLP septic rats.

### 3.4 Overexpression of miR-122-5p alleviated neuroinflammation in the PWM of CLP septic rats

**Figure 9:**
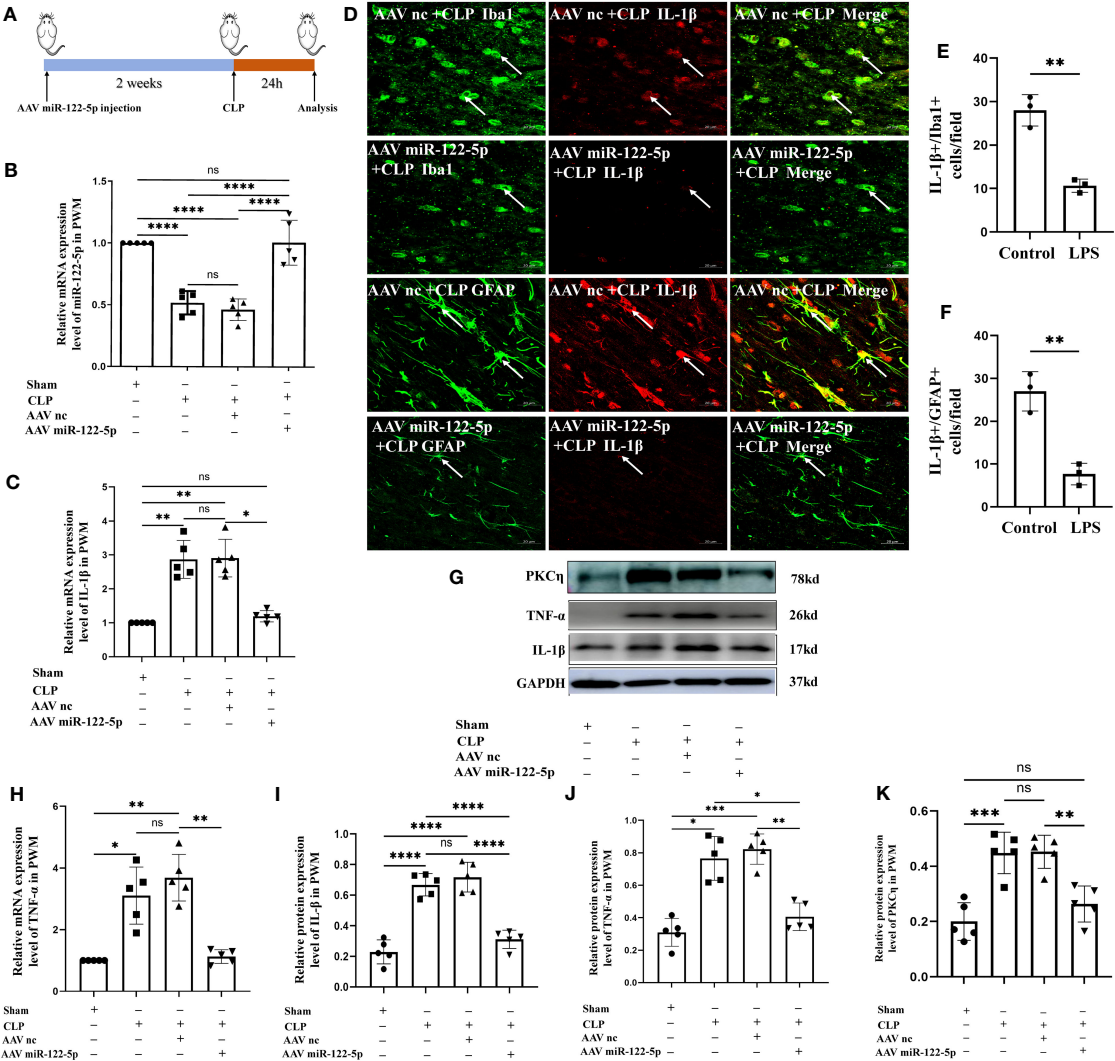
Overexpression of miR-122-5p alleviated neuroinflammation in the PWM of septic rats. **(A)** Flow chart of animal model construction. **(B)** miR-122-5p expression in the PWM of AAV miR-122-5p or AAV miR-nc injected rats at 24h after CLP as detected by RT-PCR (n=5 for each group). **(C, H)** mRNA expression of IL-1β **(C)** and TNF-α **(H)** in the PWM of AAV miR-122-5p or AAV miR-nc injected rats at 24h after CLP as detected by RT-PCR. **(D)** Immunofluorescence images of PWM showing the expression of GFP (green) or Iba1 (green) and IL-1β (red) after AAV miR-nc +CLP or AAV miR-122-5p+CLP treatment at the magnification of ×20 (n=3). The bar graph in **(E, F)** shows a significant decrease in the number of IL-1β^+^/Iba1^+^ or IL-1β^+^/GFAP^+^ cells in the PWM at 24 hours after AAV miR-122-5p+CLP in comparison with AAV miR-nc +CLP. **(G)** shows the immunoreactive bands of IL-1β (17kDa), TNF-α (26kDa), PKCη (78kDa) and GAPDH (37kDa) at 24h after CLP, AAV miR-nc +CLP, AAV miR-122-5p+CLP treatment and sham group (n=5 for each group). Bar graphs **(I–K)** show the optical density of protein expression shown in **(G)** (n=5 for each group). For statistical analysis, one-way ANOVA followed by Holm-Sidak tests was used for **(B, C, H–K)** and presented as the mean ± standard error of measurement (SEM). PWM, periventricular white matter; CLP, cecal ligation perforation. Scale bars: D, 20µm *p<0.05, **p <0.01, ***p <0.001.

To verify if enhanced miR-122-5p expression would attenuate neuroinflammation, we first explored whether intracerebroventricular injection (ICV) of miR-122-5p would reduce neuroinflammation in the PWM of CLP septic rats. AAV miR-122-5p or AAV nc were injected into left lateral ventricular. At 14 days after AAV miR-122-5p or AAV nc injection, CLP operation was performed. The rats were sacrificed at 1 day after CLP for different experiments. The flow chart was showed in the **Figure 9A**. We first examined the efficacy of AAV miR-122-5p transfection in vivo by immunofluorescence staining. GFP-tagged virus was widely co-localized in Iba1^+^ or GFAP^+^ cells in the PWM (**Supplementary Figure 3**). qPCR analysis showed that increased miR-122-5p expression was observed in the PWM of AAV miR-122-5p-injected rats when compared with that in the AAV miR-nc injected group (**Figure 9B**). The mRNA levels of IL-1β and TNF-α significantly decreased in AAV-miR-122-5p group when compared with that in AAV-miR-nc group (**Figures 9C, H**). Furthermore, the protein levels of IL-1β, TNF-α and PKCη in four groups were detected using western blotting in **Figure 9G**. The optical density of immunoreactive bands of IL-1β, TNF-α and PKCη proteins in the PWM of CLP group were increased, while miR-122-5p overexpression can reduce the expression of IL-1β (**Figures 9G, I**), TNF-α (**Figures 9G, J**) and PKCη (**Figures 9G, K**). Double immunofluorescence showed that IL-1β was colocalized with Iba1^+^ or GFAP^+^ cells. A reduction in number of IL-1β^+^/Iba1^+^ or IL-1β^+^/GFAP^+^ cells in the PWM of AAV miR-122-5p injected rats when compared with that in miR-nc AAV-injected rats (**Figures 9D–F**).

### 3.5 Knockdown of lncRNA xist expression inhibits neuroinflammation in the PWM of CLP septic rats

To validate the role of lncRNA xist in the pathogenesis of neuroinflammation in vivo, we constructed the antisense oligonucleotide (ASOs) of lncRNA xist to knock down the expression of lncRNA xist. Either the ASOs nc or ASOs lncRNA xist were microinjected into the ICV of rats. CLP operation was performed at 3days after ASOs injection. At 24h after the CLP, the rats were sacrificed and the PWM was removed for different experiments. The flow chart of specific operation was shown in **Figure 7A**. The level of lncRNA xist, which was detected by qPCR, was significantly upregulated in the PWM of CLP septic rats when compared with the corresponding controls (**Figure 7B**). The decreased lncRNA xist expression was observed in the PWM of ASOs lncRNA xist injected rat as compared with that in the ASOs nc injected group or CLP group (**Figure 7B**). However, the level of miR-122-5p was significantly upregulated in the PWM of rats in ASOs lncRNA xist group in comparison with the ASOs nc group or CLP group (**Figure 7C**). These results indicated that knockdown of lncRNA xist could increase the expression of miR-122-5p. The mRNA levels of IL-1β and TNF-α were significantly increased in the PWM of CLP septic rats when compared with the corresponding controls. Reduction in IL-1β and TNF-α mRNA level was detected in ASOs lncRNA xist group in comparison with the ASOs nc group or CLP group (**Figures 7D, E**). These results showed that knockdown of lncRNA xist could attenuate the production of proinflammatory mediators in the PWM of CLP septic rats. The immunoreactive bands of IL-1β and TNF-α protein levels appeared at approximately 17kDa and 26kDa, respectively (**Figure 7F**). The optical density of IL-1β and TNF-α protein levels were significantly increased in the PWM of rats in CLP group when compared with the controls, while it was saliently reduced in ASOs lncRNA xist group (**Figures 7G, H**). These results indicated that knockdown lncRNA xist could attenuate neuroinflammation in the PWM of CLP septic rats.

### 3.6 Mimic miR-122-5p could reduce the production of IL-1β and TNF-α in BV2 cells and astrocytes treated with LPS *in vitro*

Our study showed that miR-122-5p expression was downregulated in the PWM of CLP septic rats. Microglia and astrocytes are the main inflammatory cells in the PWM. We next examined the role of miR-122-5p in the activation of microglia and astrocytes *in vitro*. The expression of miR-122-5p was significantly suppressed in BV2 cells and astrocytes administrated with LPS in comparison with controls (**Figures 2A**, **3A**). Before transfection, the effect of transfection reagents on cell viability was detected by CCK8. The concentration of LPS and transfection reagents were 1ug/ul and 50nM, respectively. The results showed that transfection reagents including LPS, mimic-nc, mimic miR-122-5p had no effect on the viability of BV2 cells and astrocytes (**Figures 2B**, **3B**). To further elucidate whether the enhanced expression of miR-122-5p could reduce the neuroinflammation, we transfected miR-122-5p mimics or mimic nc into BV2 cells or astrocytes. Subsequently, proinflammatory mediators such as IL-1β and TNF-α were measured. The mRNA levels of IL-1β and TNF-α were significantly decreased in BV2 cells and astrocytes in LPS + mimic miR-122-5p group when compared with that in LPS group or in LPS + mimic nc group (**Figures 2C, D**, **3C, D**). Next, the protein levels of IL-1β, TNF-α and PKCη expression were also detected by western blot. Upregulation of IL-1β, TNF-α and PKCη proteins was observed in BV2 cells and astrocytes in LPS group or LPS + mimic nc group when compared with the controls (**Figures 2E–H**, **3E–H**). However, the protein levels of IL-1β, TNF-α and PKCη in mimic miR-122-5p group were saliently downregulated (**Figures 2E–H**, **3E–H**). Furthermore, double immunofluorescence showed that IL-1β expression in BV2 cells or astrocytes in the mimic miR-122-5p + LPS group was significantly decreased when compared with that in mimic nc + LPS group (**Supplementary Figures 4, 5**). Based on these results, we speculate that mimic miR-122-5p could attenuate the levels of proinflammatory mediators produced by LPS activated BV2 cells or astrocytes.

### 3.7 Upstream and downstream target genes of miR-122-5p

To further explore its mechanism that miR-122-5p is involved in the occurrence of neuroinflammation, upstream and downstream target genes of miR-122-5p were predicted by bioinformatics methods. Surprisingly, there are 101 lncRNAs at its upstream and lncRNA xist is one of these genes (**Figure 6D**). qPCR analysis also showed that lncRNA xist were significantly upregulated in BV2 cells (**Figure 4A**) and astrocytes (**Figure 4C**) in LPS group when compared with control group.

To identify the downstream target genes of miR-122-5p, we used the Starbase to screen. Results showed that PKCη mRNA has a conserved site within its 3′-UTR, which bind to miR-122-5p by complementary base pairs (**Figure 4E**). In addition, the mRNA level of PKCη was significantly upregulated in BV2 cells (**Figure 4B**) and astrocytes (**Figure 4D**) in LPS group when compared with control group.

To further confirm a targeting relationship between miR-122-5p and PKCη, the mut-type of PKCη mRNA was constructed for dual-luciferase reporter assay (**Figure 4E**). The results showed that co-transfection of mimic miR-122-5p with the wild-type prkch 3′-UTR plasmid resulted in the downregulation of luciferase activity, while this effect was reversed in 293T cells transfected with a mutated prkch 3′-UTR (**Figure 4F**). Localization of lncRNA xist expression in BV2 cells and astrocytes were detected using FISH analysis. The results showed that lncRNA xist expression was colocalized both in the cytoplasm of BV2 cells and astrocytes (**Figures 4G, H**).

### 3.8 Knockdown of lncRNA xist and PKCη attenuated the generation of IL-1β and TNF-α via NF-κB signaling pathway in BV2 cells and astrocytes administrated with LPS

To further explore the role of lncRNA xist in the production of proinflammatory mediators, the level of lncRNA xist was detected in BV2 cells and astrocytes after transfected with si-lncRNA xist. The qPCR results showed that the mRNA level of lncRNA xist were significantly reduced in BV2 cells (**Figure 8A**) and astrocytes (**Figure 8I**) in si-lncRNA xist + LPS group when compared with that in LPS group or si-lncRNA nc + LPS group. In parallel with this, the level of miR-122-5p were significantly upregulated in BV2 cells (**Figure 8B**) and astrocytes (**Figure 8J**) in si-lncRNA xist + LPS group when compared with that in si-lncRNA nc + LPS group and LPS group. An upregulation of IL-1β and TNF-α mRNA expression was observed in BV2 cells (**Figures 8C, D**) and astrocytes (**Figures 8K, L**) in LPS group or in LPS + si-lncRNA nc group when compared with that in control group. However, mRNA levels of IL-1β and TNF-α were significantly decreased in BV2 cells (**Figures 8C, D**) and astrocytes (**Figures 8K, L**) in si-lncRNA xist + LPS group in comparison with that in si-lncRNA nc + LPS group and LPS group. Furthermore, double immunofluorescence showed that IL-1β expression in BV2 cells or astrocytes in the si-lncRNA xist+LPS group was significantly decreased when compared with that in si-nc + LPS group (**Supplementary Figures 6, 7**). To further clarify the role of PKCη in the occurrence of neuroinflammation, the BV2 cells and astrocytes were transfected with si-PKCη or si-PKCη nc before LPS treatment. The immunoreactive bands of PKCη, IL-1β and TNF-α protein levels that appeared at approximately 78kDa, 17kDa and 26kDa, respectively, increased significantly in optical density in BV2 cells (**Figures 8E–H**) and astrocytes (**Figures 8M–P**) in si-PKCη nc + LPS group and LPS group as compared with the controls. However, the protein expression of PKCη, IL-1β and TNF-α was saliently inhibited in si-PKCη+ LPS group when compared with that in si-PKCη nc + LPS group or LPS group (**Figures 8E–H, M–P**). Then, we also detected the level of p-P65 and p-iKBa in BV2 cells and astrocytes after PKCη knockdown. The levels of p-P65 and p-iKBa were significantly down-regulated after si-PKCη when compared with si-PKCη nc group. The results were displayed as **Supplementary Figures 8A, C–F, B, G–J**.

## 4 Discussion

miRNAs are small noncoding RNAs whose main role is to regulate mRNA and protein levels by its degradation (28). In recent years, miRNAs have also been proven to be excellent biomarkers for diagnosis and prognosis, especially in the field of CNS diseases, due to their small size which enables them to easily cross the blood–brain barrier (BBB) compared to other biological molecules (15, 29). There are convincing evidences to suggest that miRNAs are mediators of sepsis and CNS diseases (28). The current analysis of clinical parameters of ICI patients and non-ICI patients in our study revealed the following new key findings. Firstly, miR-122-5p level was significantly decreased in CSF of ICI patients. Secondly, miR-122-5p level was negatively correlated with IL-1β or TNF-α in ICI patients. These findings suggest that miR-122-5p may be closely associated with ICI or neuroinflammation.

miRNAs in the PWM of CLP 1d rats and corresponding control were performed using high throughput sequencing in the present study. A total of 93 high expression miRNAs and 145 low expression miRNAs were identified. Among them, miR-122-5p expression was downregulated with 1.5-fold changes in CLP group. Currently, increasing evidences have confirmed that miR-122-5p was involved in sepsis-induced lung injury, myocardial injury, and spinal cord injury. Therefore, we speculate that miR-122-5p played an important role in SAN. Notably, the miR-122-5p in the PWM was significantly downregulated at 6h, 24h, 3d, and 7d after CLP. However, the level of miR-122-5p in the PWM of septic rats or in the CSF of ICI patients were not consistent with its level in the lung or myocardium. Possible causes may be attributed to differences in both organizational and cellular types, as well as differences in disease types and treatment methods. To further confirm the relationship between miR-122-5p and neuroinflammation, AAV miR-122-5p was injected into the ICV of rats. In addition, overexpression miR-122-5p could alleviate the IL-1β and TNF-α secretion in the PWM of CLP septic rats. These results demonstrated that miR-122-5p was involved in the occurrences of neuroinflammation.

The ceRNA hypothesis was first proposed in 2011 and then widely accepted in the field of non-coding RNA, and it has been described that lncRNAs can function as miRNA sponges and derepress the function of target mRNAs through competitive binding with miRNAs (30). To explore the mechanisms for miR-122-5p downregulation, bioinformatics was subsequently used to predict the upstream and downstream target genes of miR-122-5p. Therefore, we used the Starbase database to analyze the upstream target genes and found there were 101 lncRNA target genes of miR-122-5p. Moreover, lncRNA xist is the upstream target genes of miR-122-5p. Original roles of lncRNA xist is in X-chromosome dosage compensation. Accumulating evidence suggests that lncRNA xist serves as an important regulator in cell growth and development (30, 31). LncRNA xist also plays a role in septic associated lung injury, kidney injury, ischemic stroke and cerebral infarction by functioning as a competing endogenous RNA (ceRNA) (27, 30, 32). Following, the mRNA level of lncRNA xist in the PWM was detected by RT-qPCR and found that its level was upregulated in the PWM of CLP septic rats. ASOs of lncRNA xist were administered via lateral ventricle injection. The results showed that knockdown of lncRNA xist significantly decreased inflammation in the PWM of CLP rats when compared with the NC group. lncRNA XIST knockdown also could inhibit neuroinflammation by reducing expression of inflammatory cytokines including IL-1β and TNF-α. Meanwhile, after silencing lncRNA xist, the level of miR-122-5p was saliently upregulated. In addition, the CLP group with ASO nc have significant difference, this result would be associated with lateral ventricle injections. Lateral ventricle injection may cause craniocerebral injury in some degree and induce intracranial inflammation.These results demonstrate that lncRNA xist silencing significantly inhibited SAN by acting as a ceRNA and sponge miR-122-5p. However, mechanism that lncRNA xist/miR-122-5p induced neuroinflammation remains unclear.

In the CNS, neuroinflammation is characterized by release of a large amounts of inflammatory mediators, activation of glial cells, and leukocyte recruitment (10). Microglia and astrocytes are the primary immune cells in the brain (7). Microglia and astrocytes activation are considered to be a hallmark of neuroinflammation, which has been shown to contribute to the brain pathogenesis (6). We reported in our study that various inflammatory mediators, such as IL-1β and TNF-α, were released in the PWM of septic rats. Double immunofluorescence staining labeled with GFAP^+^/IL-1β^+^ or Iab1^+^/IL-1β^+^ in the PWM revealed the presence of microglia and astrocytes activation. This is consistent with the previous report that microglia might contribute to the early phase, whereas astrocytes are involved in the release of inflammatory mediators in the late phase (11).

In our study, we are dedicated to investigate the roles of microglia and astrocytes in SAN. To further investigate the molecular regulation of miR-122-5p, the level of miR-122-5p in primary astrocytes and BV2 cells were detected after LPS stimulation. As we know, activated microglia have been classified into the M1 and M2 phenotypes in pathological conditions. The M1 phenotype represents the proinflammatory cellular state, which is coupled with upregulated expression of pro-inflammatory mediators. Following, activated microglia induce the generation A1 astrocytes by secreting IL-1α, TNF-α and C1q, and that these cytokines together are necessary and sufficient to induce A1 astrocytes. Activated microglia and astrocytes produce detrimental pro-inflammatory cytokines such as IL-1β and TNF-α). In our study, the level of miR-122-5p in BV2 cells and primary astrocytes were decreased after LPS treatment. Importantly, LPS-induced inflammatory responses were reversed using miR-122-5p mimics. These results demonstrated that miR-122-5p participated in the SAN via activation of microglia and astrocytes. Then, the level of the upstream target genes lncRNA xist were detected. We found that the level of lncRNA xist was also increased in the BV2 cells and astrocytes after LPS stimulation, which was consistent with the abovementioned findings in CLP septic rats. When using the siRNA of lncRNA xist, we found that the level of lncRNA xist were both inhibited in BV2 cells and primary astrocytes, while the level of miR-122-5p were increased.

Furthermore, miRNAs target partially complementary 3′-UTR sequences of mRNAs, leading to the downregulation of protein expression. In our study, the downstream target genes of miR-122-5p were predicted and found that PKCη is a downstream target gene. Protein kinase C (PKC) is a family of protein kinases which participate in controlling the function of other proteins by phosphorylating the hydroxyl groups of the serine and threonine amino acid residues on these proteins (33). Presently, PKC are divided into the following three categories, classical PKCs, novel PKCs and atypical PKCs (33–35). The classical PKCs contain the α, βI, βII, and γ isoforms. They require Ca^2+^, 1,2-Diesteryl glycerol (DAG), and phospholipids such as phosphatidylserine for activation. The novel PKCs include the δ, ϵ, η, and θ isoforms, which require DAG but not Ca^2+^ for activation. PKCs have been associated with tumor, Alzheimer’s disease (AD), stroke and so on (33, 36, 37). As we know, PKC participates in gene regulation, including proliferation, differentiation, survival, and cell fate determination (36, 38). Different PKC isoforms have unique cellular functions and phosphorylate unique protein substrates. Many studies have already demonstrated that novel PKCs also play an important role in the inflammation. PKCδ has been reported to regulate NF-kB activation through the IKK complexes and phosphorylation of the NF-kB inhibitor IkB (39). There are few studies on PKCη in neuroinflammation (40). We demonstrated a targeted relationship between miR-122-5p and PKCη by double luciferase reporter gene assay. In addition, the miR-122-5p mimic decreased endogenous expression of PKCη in BV2 cells and astrocytes. Moreover, the miR-122-5p mimics significantly decreased the expression of PKCη in both mRNA and protein levels in LPS-stimulated BV2 cells and astrocytes. PKCη was upregulated in BV2 cells and astrocytes after LPS treatment. But it can be downregulated using the miR-122-5p mimics. Therefore, we believe that there is a targeted interaction between PKCη and miR-122-5p, and miR-122-5p is involved in the regulation of PKCη-related neuroinflammation. In our study, the levels of IL-1β and TNF-α in BV2 cells and astrocytes were significantly decreased after knockdown PKCη, which means PKCη is also involved in the neuroinflammation. These results demonstrated that miR-122-5p participated in the neuroinflammation *in vitro* and *in vivo* by promoting microglia and astrocytes activation. Studies have shown that PKCη enhanced the nuclear localization of RelA/P65 by promoting IKK activation and iKB degradation (41). In our study, we demonstrated that the level of IL-1β and TNF-α were decreased in the BV2 cells and astrocytes after knocking down PKCη by western bolt. At the same time, we also detected the level of relevant proteins in the NF-kB signaling pathway after PKCη knockdown, and found that the levels of phosphorylated p65 and p-iKBα were down-regulated after si-PKCη. This also illustrated that the PKCη was involved in the inflammatory response regulated by NF-kB signaling pathway.

Although we provide a possible mechanism in neuroinflammation, our study still has some limitations. First, the sample of ICI patients was relatively small. In future studies, we will continue to expand the sample size and build a predictive model to evaluate the prognosis of patients with SAN. Second, we have only explored the mechanism of inflammatory response at the animal and cellular levels, and there are certain differences between humans and animals, and this study can only provide certain research value for clinical practice and cannot be fully applied to clinical practice. Finally, the role of neuroinflammation in cognitive dysfunction remains unexplored.

## 5 Conclusion

**Figure 10:**
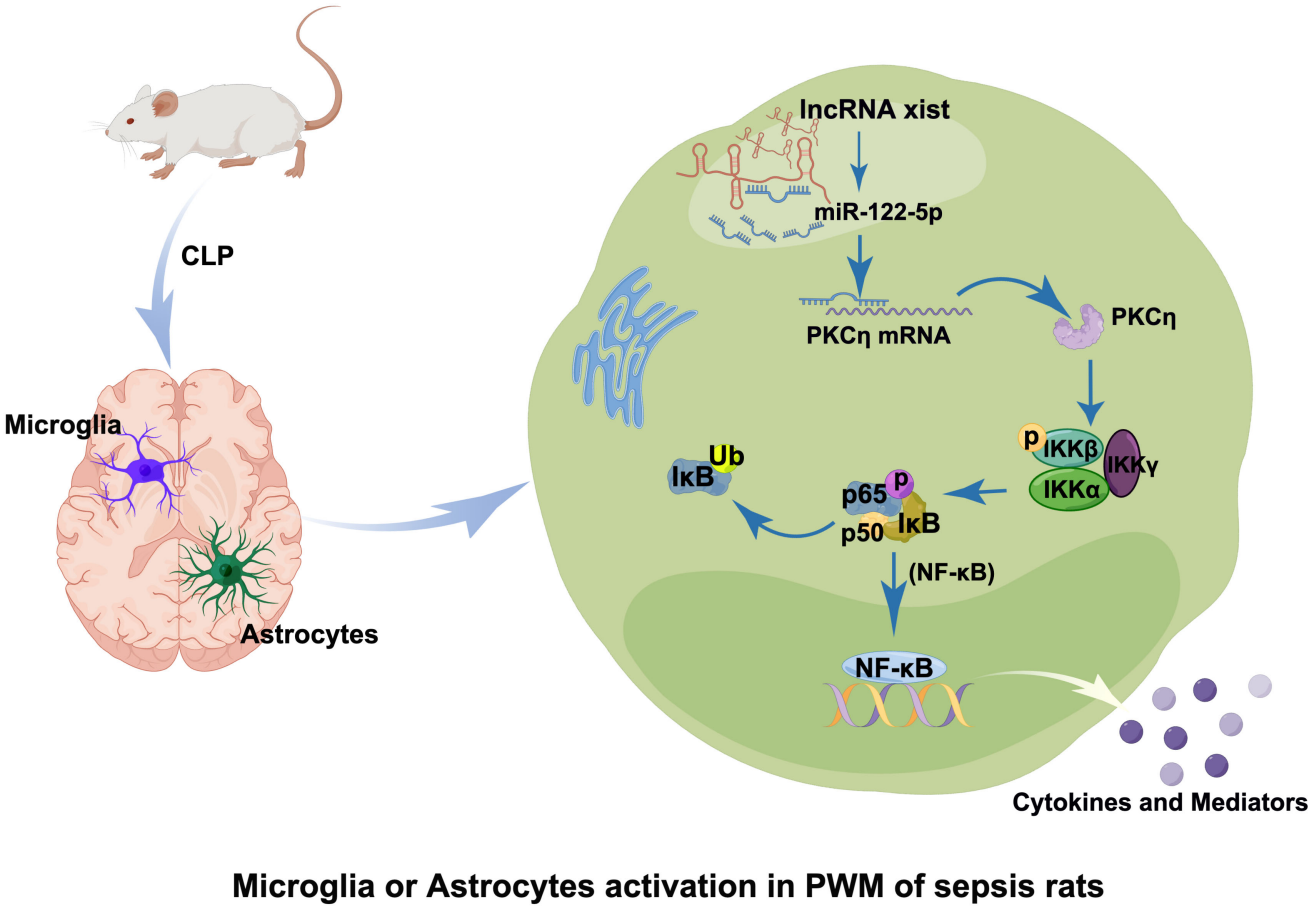
Table of Contents Image (TOCI). A schematic diagram demonstrates the cellular and molecular mechanisms associated with neuroinflammatory response in the PWM of CLP septic rats. The expression of lncRNA xist is upregulated in the cytoplasm of activated microglia and astrocytes after CLP, which would adsorb miR-122-5p as sponge gene and downregulate its expression. Reduction of miR-122-5p level may alleviate the inhibition of PKCη, which would activate NF-κB signaling pathway and promote the release of proinflammatory mediators from activated microglia and astrocytes. It would lead to the occurrence of neuroinflammation in the PWM of septic CLP rats.

This study showed that the level of miR-122-5p in the CSF of ICI patients was significantly reduced, with upregulated expression of TNF-α and IL-1β. In addition, the miR-122-5p is negatively correlated with the expression of IL-1β or TNF-α. In the PWM of CLP septic rats, microglia and astrocytes *in vivo* are activated and produced a large number of proinflammatory mediators such as IL-1β and TNF-α. Overexpression of miR-122-5p and knockdown of lncRNA xist could alleviate SAN. Furthermore, the knockdown of lncRNA xist can increase the levels of miR-122-5p and decrease the expression of PKCη. *In vitro*, lncRNA xist could induce the activation of microglial and astrocytes to produce pro-inflammatory cytokines through the miR-122-5p/PKCη pathway. Based on the current data, it can be speculated that lncRNA xist may participate in the occurrence of neuroinflammation by activating the miR-122-5p/PKCη pathway in microglia and astrocytes (**Figure 10**). This study will help develop potential therapeutic strategies to alleviate brain damage caused by neuroinflammation.

## Data availability statement

The datasets presented in this study can be found in online repositories. The names of the repository/repositories and accession number(s) can be found below: PRJNA977402 (SRA).

## Ethics statement

The studies involving humans were approved by The clinical study protocol was approved by the ethics committee at Guangdong provincial People’s Hospital (approval NO. KY-Q-2022-176-02. The studies were conducted in accordance with the local legislation and institutional requirements. The participants provided their written informed consent to participate in this study. The animal study was approved by Animal ethics were approved by the Guangdong provincial People’s Hospital (approval ID: SYXK2012-0081. The study was conducted in accordance with the local legislation and institutional requirements.

## Author contributions

Designed and conceived the experiments: YD and CC. Performed the experiments and analyzed the data: HW, YCL, and SJ. Contributed materials/reagents/analysis tools: HW, SJ, NL, QZ, QL, YYL, ZC. Wrote the paper: HW, YD. All authors contributed to the article and approved the submitted version.
