# Supplementary figures and images for: LncRNA xist regulates sepsis associated neuroinflammation in the periventricular white matter of CLP rats by miR-122-5p/PKCη Axis
Source: Front Immunol. 2023 Dec 5;14:1225482. doi: 10.3389/fimmu.2023.1225482 (PMC10728298; doi:10.3389/fimmu.2023.1225482)

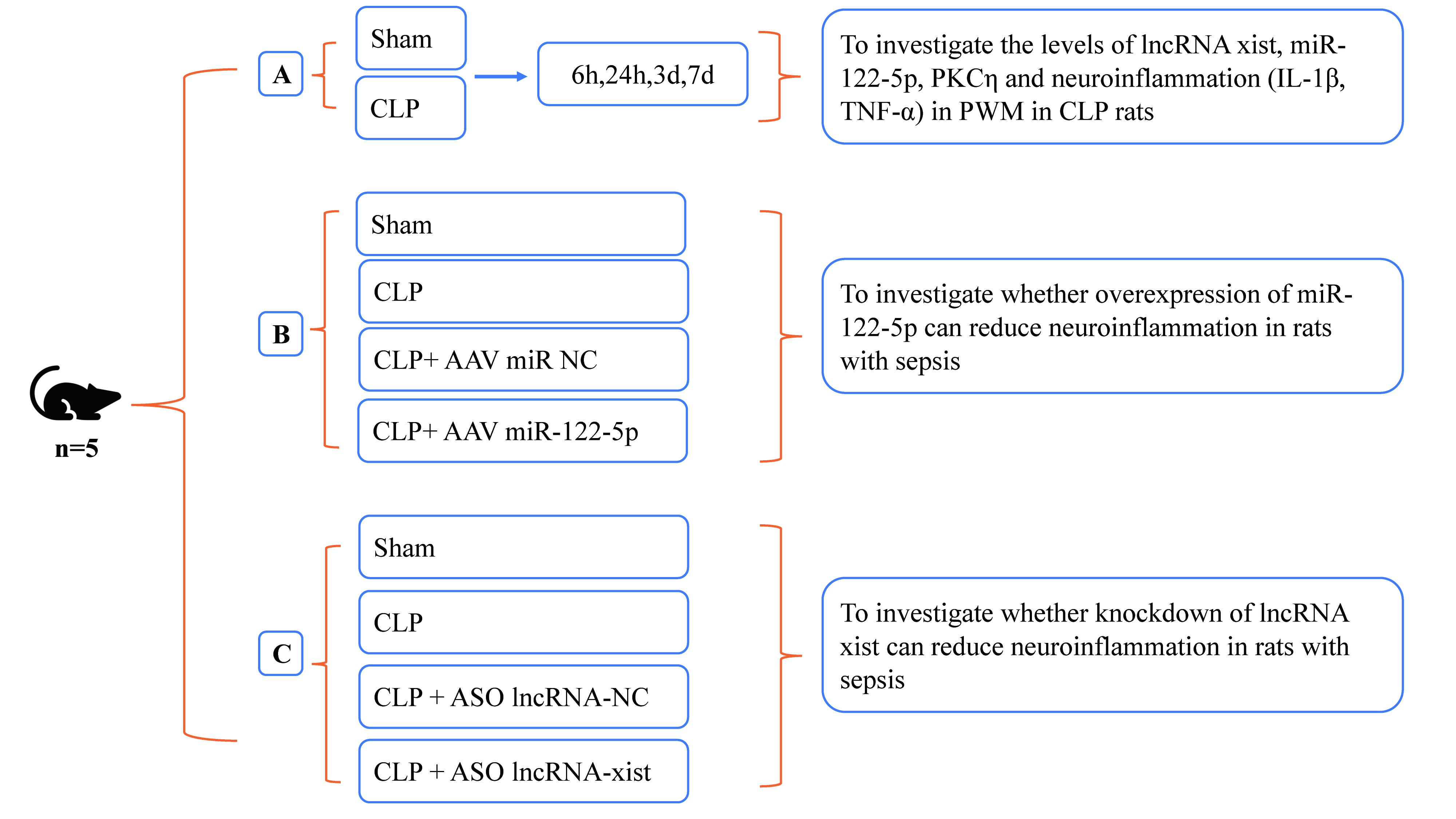

Supplement: Supplementary file 1 [file DataSheet_1.zip › Supplementary Figure 1.TIF]

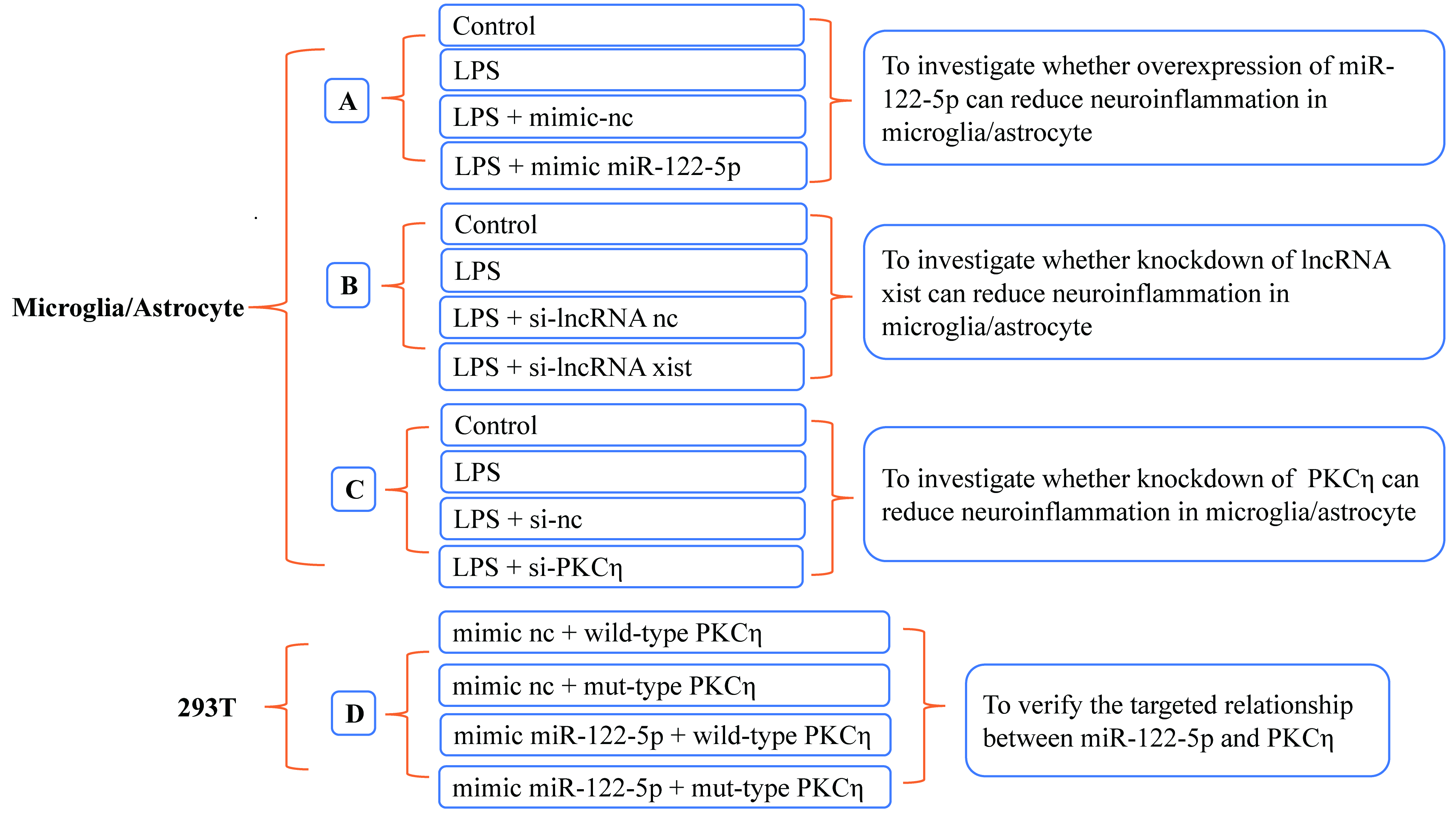

Supplement: Supplementary file 1 [file DataSheet_1.zip › Supplementary Figure 2.TIF]

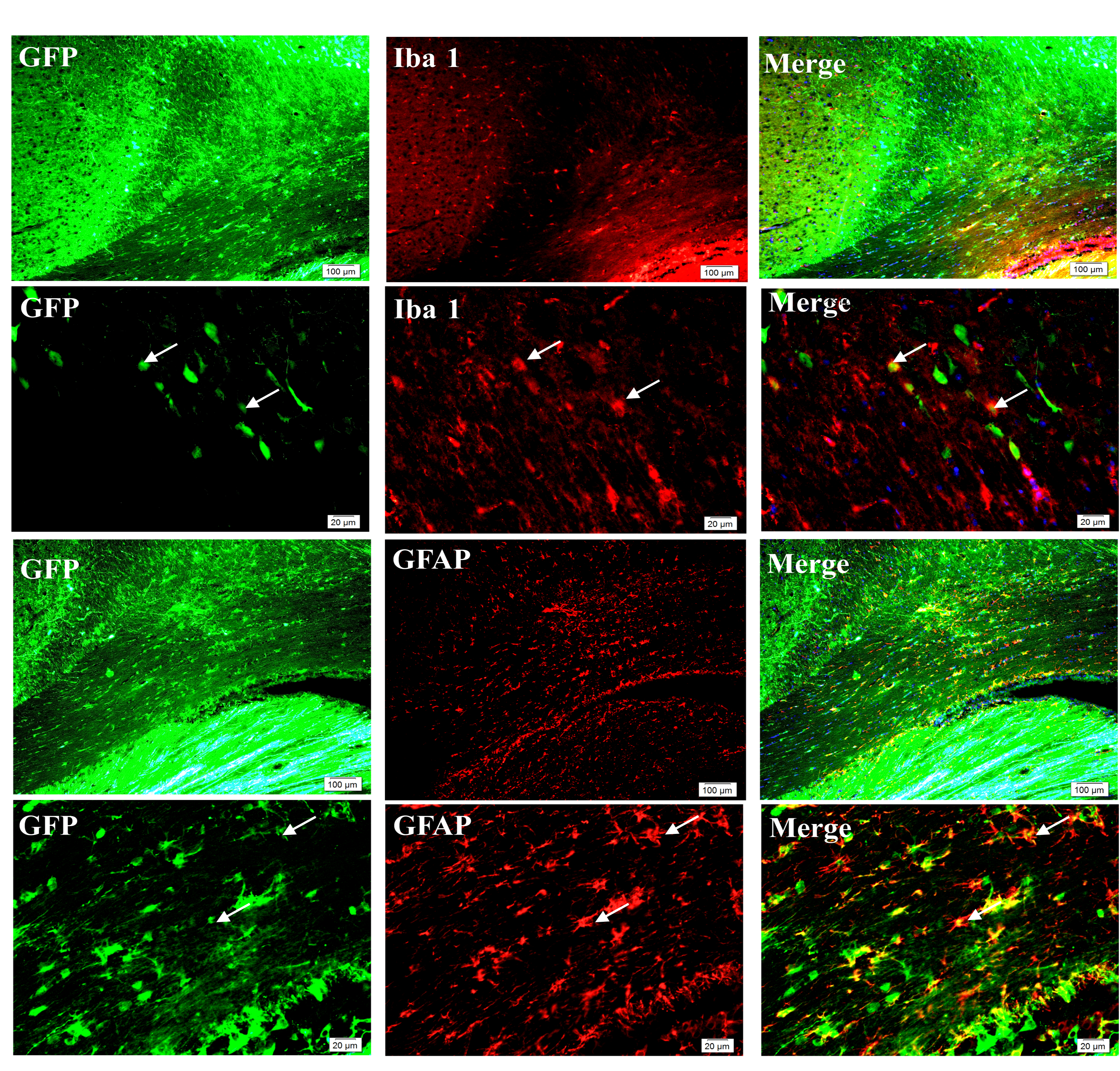

Supplement: Supplementary file 1 [file DataSheet_1.zip › Supplementary Figure 3.TIF]

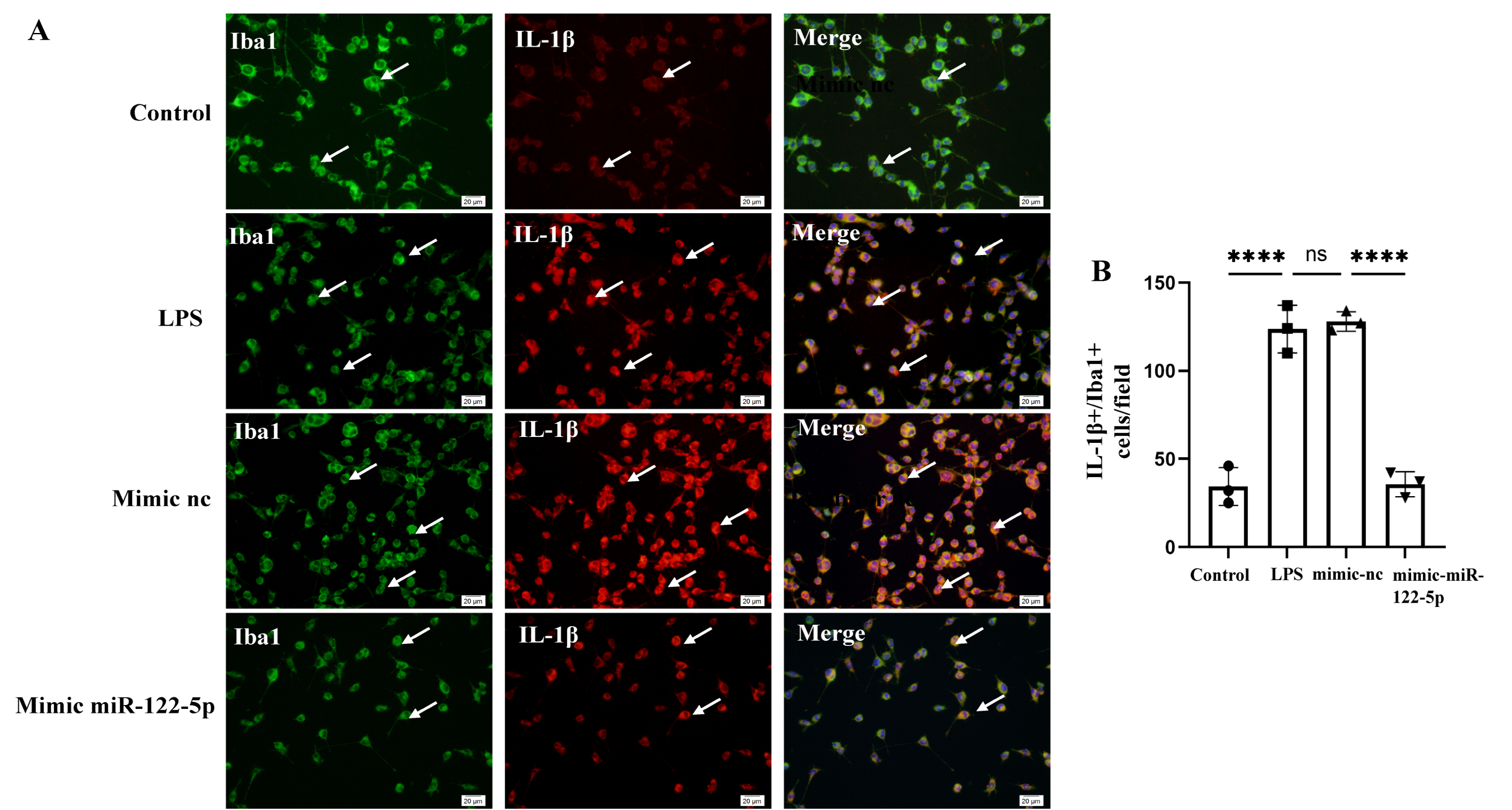

Supplement: Supplementary file 1 [file DataSheet_1.zip › Supplementary Figure 4.TIF]

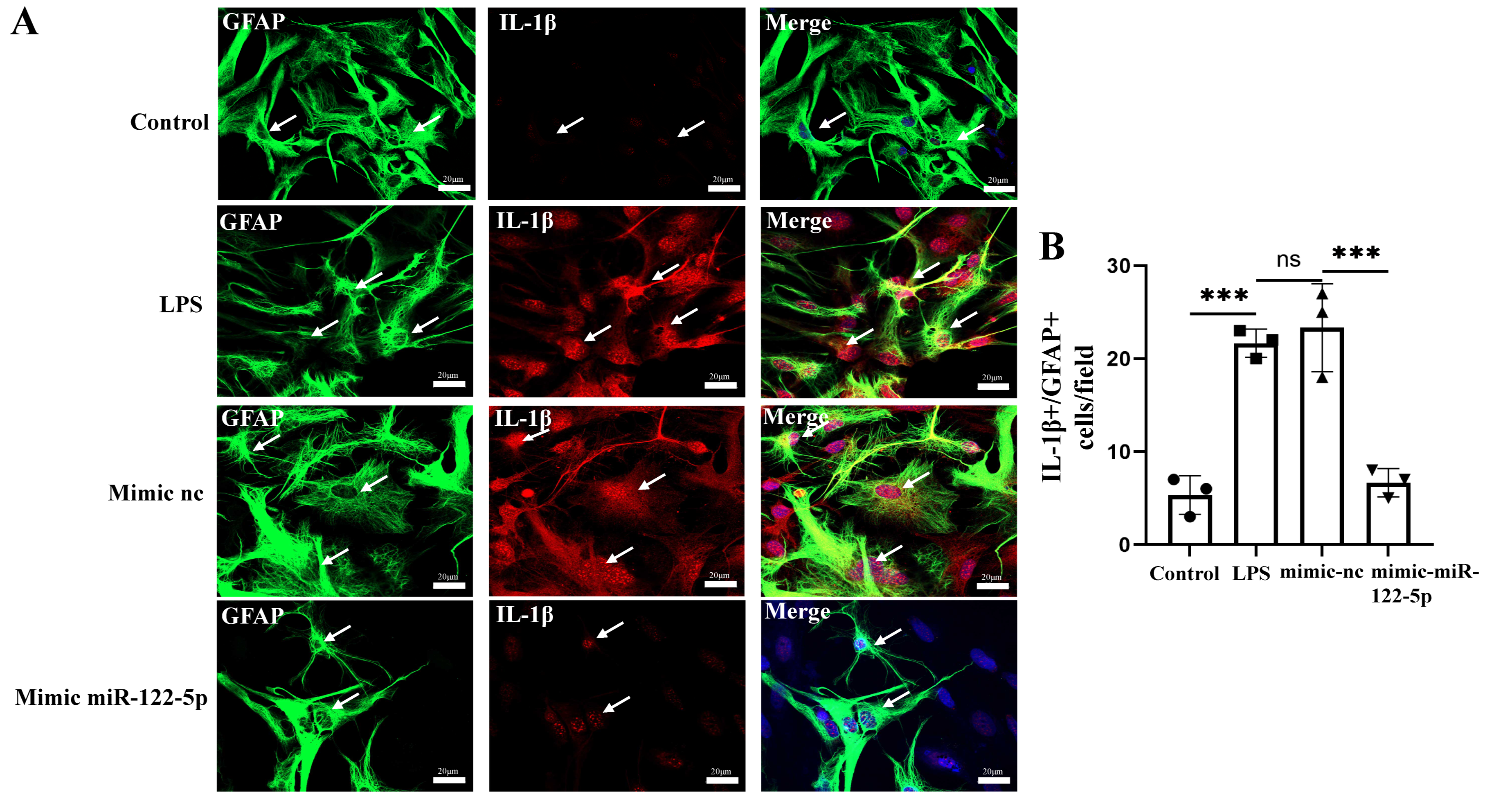

Supplement: Supplementary file 1 [file DataSheet_1.zip › Supplementary Figure 5.TIF]

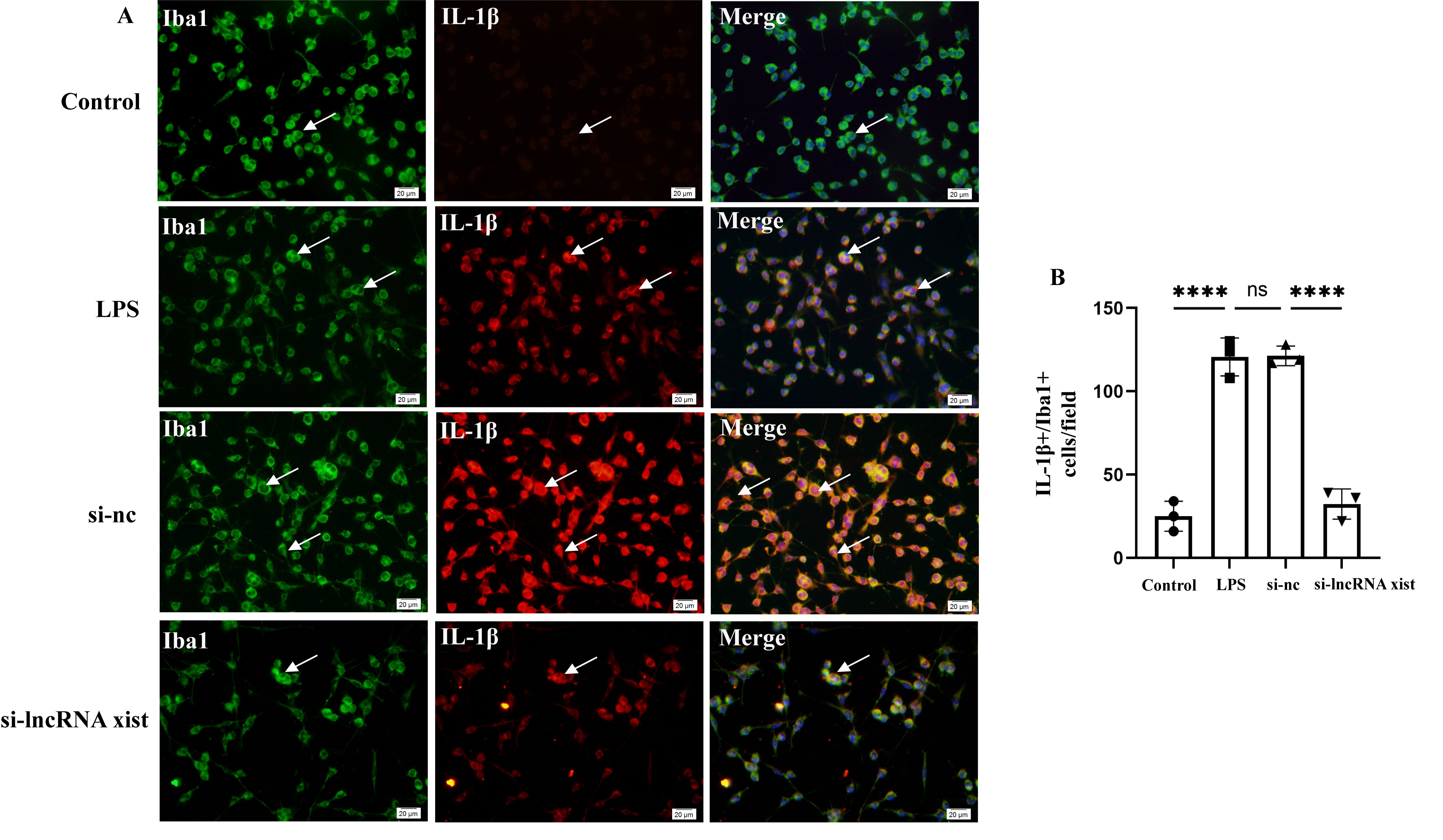

Supplement: Supplementary file 1 [file DataSheet_1.zip › Supplementary Figure 6.TIF]

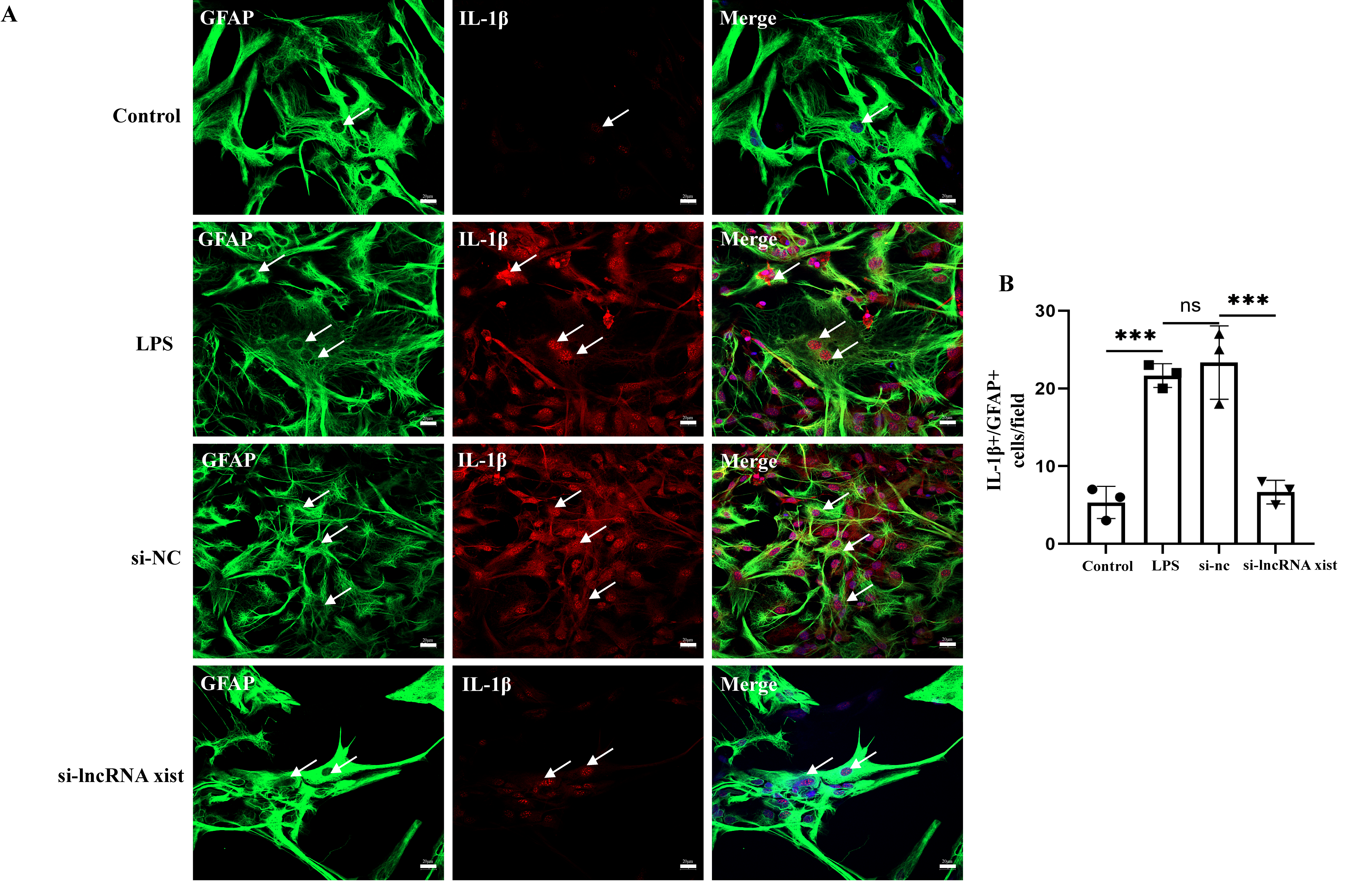

Supplement: Supplementary file 1 [file DataSheet_1.zip › Supplementary Figure 7.TIF]

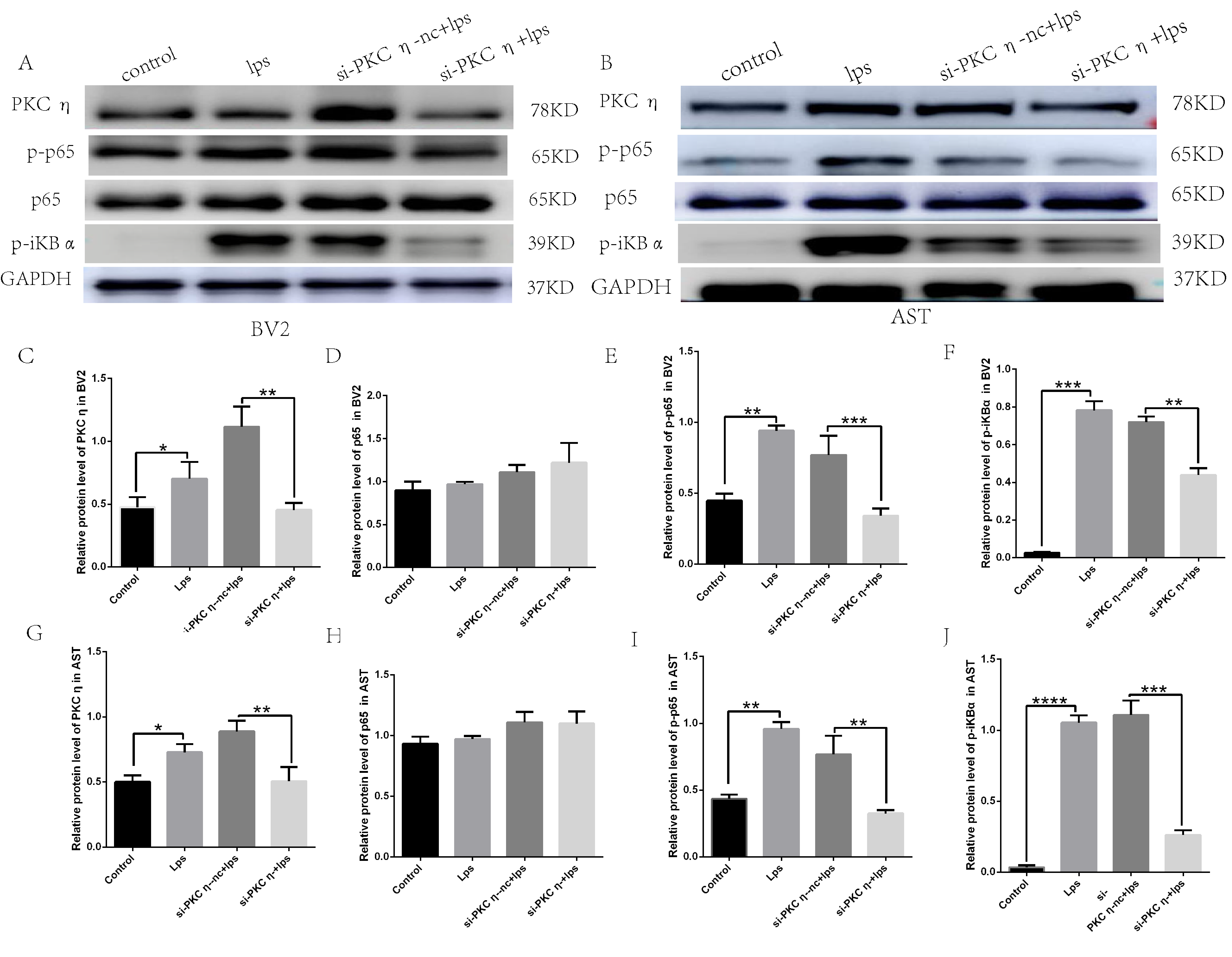

Supplement: Supplementary file 1 [file DataSheet_1.zip › Supplementary Figure 8.TIF]
