# Supplementary material for: LncRNA xist regulates sepsis associated neuroinflammation in the periventricular white matter of CLP rats by miR-122-5p/PKCη Axis
Source: Front Immunol. 2023 Dec 5;14:1225482. doi: 10.3389/fimmu.2023.1225482 (PMC10728298; doi:10.3389/fimmu.2023.1225482)
Supplement: Supplementary file 2 [file DataSheet_2.pdf]

Supplementary Material

LncRNA xist regulates sepsis associated neuroinflammation in the periventricular white matter of CLP rats by miR-122-5p/PKCη Axis

Huifang Wang[1‡], Yichen Li[1‡], Shuqi Jiang[1‡], Nan Liu[1,2], Qiuping Zhou[1], Qian Li[1,3], Zhuo Chen[1,2], Yiyan Lin[1,3], Chunbo Chen[1*†], Yiyu Deng[1*]

[1]Department of Intensive Care Medicine, Guangdong Provincial People's Hospital (Guangdong Academy of Medical Sciences), Southern Medical University, Guangzhou, China.

[2]Department of Critical Care Medicine, Guangdong Provincial People's Hospital, School of Medicine South China University of Technology, Guangzhou, China.

[3]The Second School of Clinical Medicine, Southern Medical University, Guangzhou, People's Republic of China.

*** Correspondence**
Yiyu Deng
yiyudeng666@163.com

Chunbo Chen

gghccm@163.com

[†]Present address

Chunbo Chen,

Department of Critical Care Medicine,Shenzhen People's Hospital, The Second Clinical Medical College of Jinan University, The First Affiliated Hospital of Southern University of Science and Technology, Shenzhen, China

[‡] These authors contributed equally to this work and share first authorship

Keywords: lncRNA xist, miR-122-5p, sepsis, microglia, astrocytes, sepsis associated neuroinflammation, intracranial infection

Supplementary Figures

Supplementary Figure 1 Experimental group diagram of animals in this study.

To investigate the levels of lncRNA xist, miR-122-5p, PKCη, IL-1β and TNF-α in the PWM in CLP rats. The rats were randomly divided into sham and CLP group. To investigate whether overexpression of miR-122-5p can reduce neuroinflammation in rats with sepsis, rats were divided into 4 groups: Sham, CLP, AAV miR-NC + CLP, and AAV miR-122-5p +CLP. To investigate whether knockdown of lncRNA xist can reduce neuroinflammation in rats with sepsis, rats were divided into 4 groups: Sham, CLP, ASO lncRNA-NC + CLP, and lncRNA-xist ASO +CLP.

Supplementary Figure 2 Experimental group diagram of astrocytes and BV2 in this study.

To investigate whether overexpression of miR-122-5p can reduce neuroinflammation in microglia/astrocyte, cells were treated with mimic-miR-122-5p or mimic-nc, then divided into four groups: control group, LPS group, mimic-nc + LPS group and mimic miR-122-5p+LPS group. To investigate whether knockdown of lncRNA xist can reduce neuroinflammation in microglia/astrocyte, cells were cultured and treated with si-lncRNA xist, then divided into four groups: control group, LPS group, si-lncRNA nc + LPS group and si-lncRNA xist + LPS group. To investigate whether knockdown PKCη can reduce neuroinflammation in microglia/astrocyte, cells were administrated with si-RNA, then divided into four groups: control group, LPS group, si-nc+LPS group and si-PKCη+LPS group.

Supplementary Figure 3 Transfection efficiency of recombinant adeno-associated virus 9 (AAV9) in the PWM of septic CLP rats.

The AAV9 of miR-122-5p were injected into the left lateral ventricle. Double immunofluorescence staining showed the colocalized expression of GFP (green) and Iba1/GFAP (red) after AAV9 injection at the magnification of ×20. The results showed that the AAV9 may be transfected into the microglia and astrocytes with high transfection efficiency.

Supplementary Figure 4 Mimic miR-122-5p can alleviate expression of IL-1β in BV2 cells administrated with LPS *in vitro*.

Double immunofluorescence staining shows the distribution of Iba1 (green) and IL-1β (red) immunoreactive microglial cells after treatment with LPS or LPS + mimic miR-122-5p or LPS +

mimic nc and their matching controls. Co-localized expression of Iba1 and IL-1β could be seen in **Panel A**. The bar graph in **Panel B** shows a significant increase in the number of IL-1β^{+}/Iba1^{+} cells after LPS or LPS + mimic nc in comparison with their corresponding controls or LPS + mimic miR-122-5p. Note mimic miR-122-5p could decrease the expression of IL-1β in microglia induced by LPS.

Supplementary Figure 5 Mimic miR-122-5p alleviate expression of IL-1β in astrocytes administrated with LPS *in vitro*.

Double immunofluorescence staining shows the distribution of GFAP (green) and IL-1β (red) immunoreactive astrocytes after LPS, mimc nc + LPS, mimic miR-122-5p + LPS treatment and their matching controls. Co-localized expression of GFAP and IL-1β could be seen in **Panel A**. The bar graph in **Panel B** shows a significant increase in the number of IL-1β^{+}/GFAP^{+} cells after LPS or mimc nc + LPS in comparison with mimic miR-122-5p + LPS or their corresponding controls. Note mimic miR-122-5p could downregulate the expression of IL-1β in astrocytes induced by LPS.

Supplementary Figure 6 Knockdown lncRNA xist can downregulate expression of IL-1β in BV2 cells treated with LPS.

Double immunofluorescence staining shows the distribution of Iba1 (green) and IL-1β (red) immunoreactive BV2 cells after LPS or si-nc + LPS or si-lncRNA xist + LPS treatment and their matching controls. Co-localized expression of Iba1 and IL-1β could be seen in **Panel A**. The bar graph in **Panel B** shows a significant increase in the number of IL-1β^{+}/ Iba1^{+} cells after LPS or si-nc + LPS in comparison with si-lncRNA xist + LPS or their corresponding controls. Note si-lncRNA xist could downregulate the expression of IL-1β in BV2 cells induced by LPS.

Supplementary Figure 7 Knockdown lncRNA xist can decrease expression of IL-1β in astrocytes administrated with LPS.

Double immunofluorescence staining shows the distribution of GFAP (green) and IL-1β (red) immunoreactive astrocytes after LPS or si-nc + LPS or si-lncRNA xist + LPS treatment and their matching controls. Co-localized expression of GFAP and IL-1β could be seen in **Panel A**. The bar graph in **B** shows a significant increase in the number of IL-1β^{+}/GFAP^{+} cells after LPS or si-nc + LPS in comparison with si-lncRNA xist + LPS or their corresponding controls. Note si-lncRNA xist could downregulate the expression of IL-1β in astrocytes induced by LPS.

Supplementary Figure 8 Knockdown PKCη can reduce the level of p-ikbα and p-p65 in astrocyte and BV2 cells.

A-B. Panel **A and B** shows the immunoreactive bands of PKCη (78kDa), p65 (65kDa), p-p65(65kDa) and p-iKbα (39kDa) and GAPDH (37kDa) after treatment with LPS, si-nc+LPS, si-PKCη+LPS, and the corresponding control (n=3 for each group) in BV2 cells and astrocytes. Bar graphs (**C-J**) show the optical density of protein expression shown in Panel **A and B** (n=3 for each group). For statistical analysis, one-way ANOVA followed by Holm-Sidak tests was used and presented as the mean ± standard error of measurement (SEM). *$p<0.05$, ** $p<0.01$, *** $p<0.001$

Supplementary Table 1. Primary antibodies used in experiments

The antibody name, species, manufacturers and concentrations were included.
