# Supplementary material for: LncRNA xist regulates sepsis associated neuroinflammation in the periventricular white matter of CLP rats by miR-122-5p/PKCη Axis
Source: Front Immunol. 2023 Dec 5;14:1225482. doi: 10.3389/fimmu.2023.1225482 (PMC10728298; doi:10.3389/fimmu.2023.1225482)
Supplement: Supplementary file 3 [file Table_1.docx]

**Supplementary Table 1** **Primary antibodies used in experiments**

| Antibody | Host | Company | Cat. No. | Application (Concentration) |
| --- | --- | --- | --- | --- |
| IL-1β | Rabbit | Abcam | ab9722 | WB (1:1000)/IF (1:200) |
| TNF-α | Rabbit | Abcam | ab66579 | WB (1:1000)/IF (1:200) |
| PKC-Eta | Rabbit | Lifespan | LSC26203-100 | WB (1:1000) |
| Iba1 | Rabbit | Abcam | ab178846 | IF (1:200) |
| p-p65 | Rabbit | CST | 3033S | WB (1:1000) |
| p65 | Rabbit | CST | 8242S | WB (1:1000) |
| p-iKbα | Rabbit | CST | 9246S | WB (1:1000) |
| GFAP | Rabbit | Abcam | ab7260 | IF (1:200) |
| GAPDH | Rabbit | Abcam | ab181602 | WB (1:3000) |
